# Impact of Orientational Glass Formation and Local Strain on Photo-Induced Halide Segregation in Hybrid Metal-Halide Perovskites

**DOI:** 10.1021/acs.jpcc.1c03169

**Published:** 2021-06-30

**Authors:** Tim W. J. van de Goor, Yun Liu, Sascha Feldmann, Sean A. Bourelle, Timo Neumann, Thomas Winkler, Nicola D. Kelly, Cheng Liu, Michael A. Jones, Steffen P. Emge, Richard H. Friend, Bartomeu Monserrat, Felix Deschler, Siân E. Dutton

**Affiliations:** † Cavendish Laboratory, University of Cambridge, J. J. Thomson Avenue, Cambridge CB3 0HE, U.K.; ‡ Walter Schottky Institut and Physik Department, Technische Universität München, Am Coulombwall 4, 85748 Garching, Germany; § Department of Physics and Astronomy, Aarhus University, 8000 Aarhus C, Denmark; ∥ Department of Chemistry, University of Cambridge, Lensfield Road, Cambridge CB2 1EW, U.K.; ⊥ Department of Materials Science and Metallurgy, University of Cambridge, 27 Charles Babbage Road, Cambridge CB3 0FS, U.K.

## Abstract

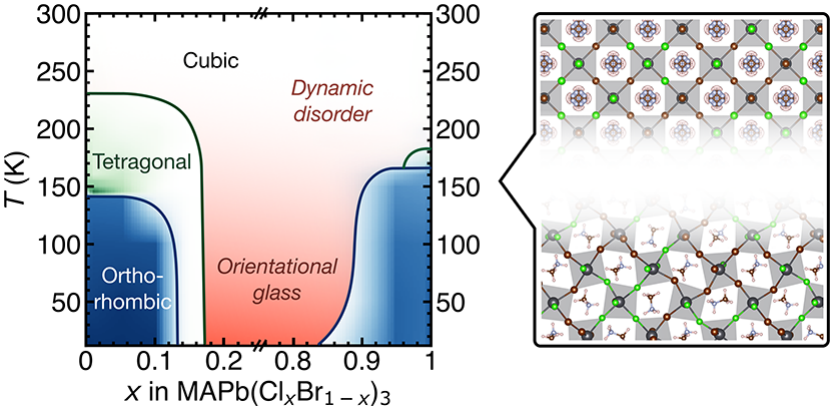

Band gap tuning of hybrid metal–halide perovskites by halide substitution holds promise for tailored light absorption in tandem solar cells and emission in light-emitting diodes. However, the impact of halide substitution on the crystal structure and the fundamental mechanism of photo-induced halide segregation remain open questions. Here, using a combination of temperature-dependent X-ray diffraction and calorimetry measurements, we report the emergence of a disorder- and frustration-driven orientational glass for a wide range of compositions in CH_3_NH_3_Pb(Cl_*x*_Br_1–*x*_)_3_. Using temperature-dependent photoluminescence measurements, we find a correlation between halide segregation under illumination and local strains from the orientational glass. We observe no glassy behavior in CsPb(Cl_*x*_Br_1–*x*_)_3_, highlighting the importance of the A-site cation for the structure and optoelectronic properties. Using first-principles calculations, we identify the local preferential alignment of the organic cations as the glass formation mechanism. Our findings rationalize the superior photostability of mixed-cation metal–halide perovskites and provide guidelines for further stabilization strategies.

## Introduction

Mixed–halide hybrid perovskites MAPbX_3_ (X = Cl, Br, I) (MA = methylammonium, CH_3_NH_3_) are promising materials for light absorption in tandem solar cells and light emission in light-emitting diodes.^1,2^ The power conversion efficiency of hybrid perovskite solar cells has recently exceeded 25%, rivalling single crystalline silicon technology.^3^ Their high performance is enabled by a large absorption coefficient,^4^ tunable band gap,^5−7^ long carrier diffusion lengths,^8^ low exciton binding energy^9^ and exceptional defect tolerance.^10^ These properties, in combination with their scalable solution processability and low cost, are unparalleled by any other thin film optoelectronic material. The interplay between the organic and inorganic constituents of this system is at the heart of its unique properties. In particular, the dynamic disorder of the organic sublattice, which is not found in traditional inorganic semiconductors, has received widespread attention in the community.^11^ It has long been known that the structure and properties of perovskites depend sensitively on their composition and disorder and this is no different in their hybrid counterparts. For example, it has been shown that compositional disorder on the cation site can trigger anomalous glassy behaviour^12,13^ and geometric frustration.^14,15^ However, the effect of halide substitution on the complex molecule–cage interactions and their implications on the crystal structure and optoelectronic properties has remained largely unexplored. Developing a detailed understanding of these structure–property relationships for mixed-halide hybrid perovskites is necessary to address halide segregation under illumination, a key challenge toward commercial applications of this material in solar cells.^16^ Previous reports have investigated the effects of stoichiometry,^17^ crystallinity,^18^ crystal structure,^19^ vacancies,^20^ temperature,^21^ and illumination intensity^22,23^ on this phenomenon. Recently, transient polaronic strain gradients originating from photo-excited charge carriers have been proposed as the main driving mechanism.^23,24^ For low excitation densities, polaronic strain gradients from charge carriers localized in low band gap regions drive the migration of halides. At high excitation densities, neighboring polaronic strain fields start to overlap, homogenizing the energetic landscape and allowing for entropic remixing of the halides. However, this model does not explain the superior stability of certain mixed-halide compositions, and a comprehensive picture on the role of local static strain is still missing. Here, we investigate the effect of halide substitution on the structure and optoelectronic properties of MAPb(Cl_*x*_Br_1–*x*_)_3_, by combining temperature-dependent X-ray diffraction (XRD), calorimetry, density functional theory (DFT) calculations, and photoluminescence (PL) spectroscopy. We uncover a suppression of the phase transitions accompanied by a spontaneous local static strain in a wide range of mixed-halide compositions, whereby the high symmetry room temperature structure is anomalously retained down to low temperature. We hypothesize that the relatively weak noncovalent interactions between the organic cations and the surrounding disordered halide distribution in the inorganic cage become dominant at low temperatures and drive the preferential alignment of the MA cations, leading to the formation of an orientational glass on the MA sublattice. The resulting incompatibility between the orientation of neighboring MA cations inhibits the collective tilts and distortions of the inorganic octahedra, suppressing the phase transitions. Using temperature-dependent PL measurements, we find that the persistence of local strains above the glass transition leads to photo-induced halide segregation, providing new evidence for the importance of static strain for the stability of mixed-halide hybrid perovskites.

## Methods

### Sample Preparation

Hybrid metal–halide perovskite powders are synthesized using a solid-state method. The precursor materials methylamine hydrochloride (MACl, 99%, Alfa Aesar), lead(II)chloride (PbCl_2_, 99.999%, Alfa Aesar), methylammonium bromide (MABr, Greatcell Solar), and lead(II)bromide (PbBr_2_, 99.998%, Alfa Aesar) were stored under a dry inert atmosphere in a glovebox (Argon, H_2_O < 0.5 ppm, O_2_ < 0.5 ppm). All solid-state reactions are carried out under the same atmosphere. For the end members, equimolar amounts of PbX_2_ and MAX (X = Cl and Br) are ground together using pestle and mortar for 30 min until a fine and homogeneously colored powder is obtained. In order to avoid decomposition of the precursors and the final product around 200 °C,^25^ the samples are reacted at 110 °C for 48 h. Mixed-halide hybrid perovskites are obtained by mixing the appropriate molar ratio of the resulting MAPbX_3_ end members and using the same reaction protocol. All-inorganic perovskite powders are synthesized using a solid-state method. For the end members, equimolar amounts of PbX_2_ and CsX (X = Cl and Br) were ground together using pestle and mortar for 30 min until a fine and homogeneously colored mixture was obtained. The powders were dried in a vacuum oven at <10^–2^ mbar for 48 h before they were sealed in evacuated quartz ampoules. The samples were then heated to 610 °C over the course of 12 h for a duration of 6 h, and then were allowed to cool down to room temperature following the previous literature reports.^12,26−28^ The samples were reground and pressed into pellets for an annealing step under an ambient atmosphere at 325 °C for 12 h. For the mixed-halide inorganic perovskites, equimolar amounts of the end members were ground together using a mortar and pestle and pressed into pellets. The pellets were annealed in an ambient atmosphere at 325 °C for 12 h. All samples were stored inside an Ar glovebox before measurement.

### Powder XRD

Powder XRD was performed using a Bruker D8 Discover diffractometer with Cu Kα1,2 radiation (λ = 1.541 Å). Low-temperature measurements were carried out using an Oxford Cryosystems PheniX stage. The samples were first cooled to *T* = 12 K with a rate of 360 K/h under a pressure of <10^–4^ mbar. Measurements were then performed upon heating between *T* = 12 K and *T* = 300 K. Spectra were collected with an angular range 2θ from 10 to 80° and a resolution of Δθ = 0.01022° with 0.5 s integration time per step, resulting in a total scan time of 1 h. Lattice parameters and microstrains are extracted from the diffraction patterns in TOPAS^29^ using the Le Bail method^30^ with the *Pm*3̅*m* space group for the room temperature measurements and the *Pnma* space group for all lower temperatures. A Cu phase was included in the refinements to account for the contribution from the copper sample stage.

### Heat Capacity

Heat capacity measurements were carried out on a Quantum Design Physical Property Measurement System (PPMS) using the two-tau model relaxation technique. Typically, a small amount of Apiezon N grease (for measurements below *T* = 300 K) or H grease (for measurements above *T* = 300 K) was deposited on the sample platform. First, a background (addenda) is measured over the desired temperature range. Then, a piece of the pressed sample pellet (5–15 mg) is carefully positioned on the platform and the measurement is carried out. The sample pellets were pressed inside an Ar glovebox at 0.3 GPa for 5 min.

### DFT Calculations

Electronic wave functions were expanded in a plane wave basis with an energy cutoff of 400 eV, and the core–valence interaction was treated by the projector-augmented wave method,^31,32^ and the exchange correlation functional was approximated by PBEsol.^33^ As all calculations are performed on large disordered supercells (∼24 Å × 24 Å × 23 Å), only the Γ-point of the Brillouin zone was sampled. Atomic positions were relaxed until the residual forces were <0.01 eV/Å. Van der Waals dispersion forces were included by Grimme’s DFT-D3 method.^34^

### Photoluminescence

Low-temperature PL measurements were carried out using two different experimental setups. The first setup (Exp1) is a custom-built flange adapter for a Quantum Design PPMS with an optical feedthrough. A cage system with mounts for optics is attached to the flange. A fiber-coupled excitation source (PicoQuant PDL 800-D laser driver with LDH-D-C-405 laser head) is attached to the top of the cage system. The laser beam outcouples through a fiber collimator and travels through a dichroic mirror (Thorlabs DMSP425R) and fused silica wedge window down the PPMS bore. It is focused on a sample using a 0.5 inch diameter lens mounted in a lens tube on top of the sample holder. The powder sample is deposited on high vacuum grease on top of the copper sample holder, which is in direct contact with the PPMS thermal bath. PL is collected with the same lens and directed out of the PPMS bore onto the dichroic mirror and is focused on a fiber incoupler which is connected to a spectrometer (ThorLabs CCS200/M). The second setup (Exp2) comprises a femtosecond Ti:Sapphire laser and a Janis cryostat. A diode laser (Coherent Verdi, 18 W) is used to pump an ultrafast oscillator (Coherent Mira, 76 MHz) and amplifier (Coherent RegA). The amplifier is seeded by 800 nm pulses from the oscillator and outputs pulses of 800 nm light at a repetition rate of 250 kHz. The pulses are focused onto a β-barium borate crystal for second harmonic generation of 400 nm pulses. The output is collimated and passes through a neutral density filter wheel (Thorlabs NDM4/M) to control the power. The beam is then focused onto the sample inside the cryostat (Janis SHI-4-2 optical cryostat system with a LakeShore model 335 temperature controller and model TS-85-D turbo pump). The powder samples are mounted on high vacuum grease on top of a glass coverslip, which is in contact with the cryostat coldfinger through a thin layer of high vacuum grease. PL from the sample is collected using the focusing lens and is directed to a spectrometer (Andor Solis) connected to an iCCD (Andor) via a dichroic mirror (450 nm longpass). In both experiments, the samples were allowed to first settle at the lowest temperature for 1 h, after which measurements were then taken upon warming up to room temperature. The samples were exposed to normal ambient lighting conditions during their synthesis and storage, prior to the PL measurements.

## Results and Discussion

### Suppression of Phase Transitions in Mixed-Halide Hybrid Perovskites

**Figure 1:**
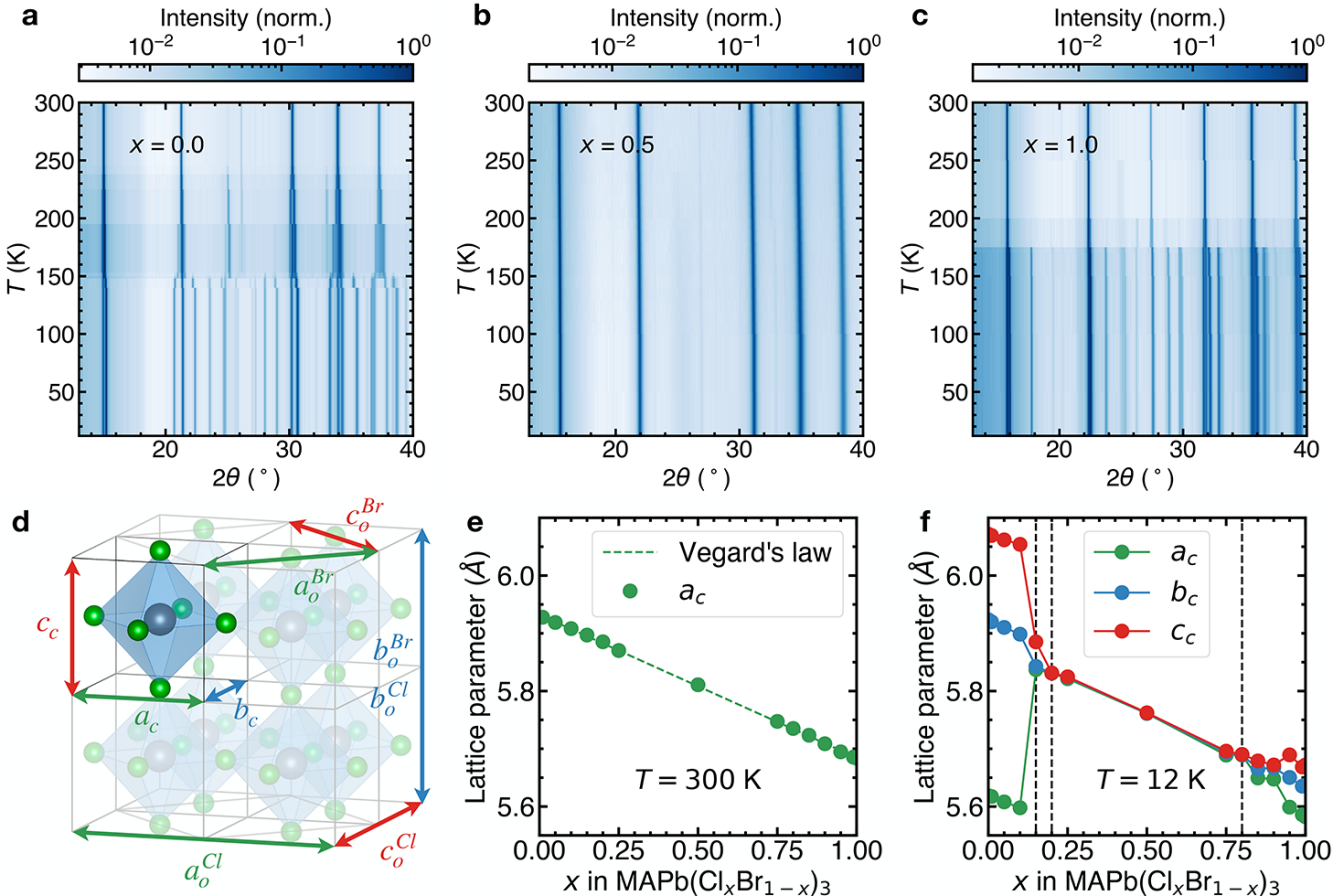
XRD and structural analysis of MAPb(Cl_*x*_Br_1–*x*_)_3_. Temperature-dependent XRD data from *x* = 0 (a), *x* = 0.5 (b), and *x* = 1 (c) compositions of the mixed-halide perovskite series. Diffraction intensity is normalized for each temperature and presented on a pseudo-color log-scale to better visualize the low intensity peaks. (d) Archetypal crystal structure of MAPbX_3_ (Pb = gray spheres, X = green spheres, MA has been omitted for clarity) with the corner-sharing octahedra depicted in blue, showing the relation between the cubic (*a*_c_, *b*_c_, and *c*_c_) and orthorhombic (*a*_o_^X^, *b*_o_^X^, and *c*_o_^X^) lattice parameters of MAPbCl_3_ (denoted by superscript X = Cl) and MAPbBr_3_ (denoted by superscript X = Br). (e) Cubic lattice parameter *a*_c_ as a function of composition *x* at *T* = 300 K, showing good agreement with Vegard’s law^36^ (dashed green line). (f) Pseudocubic lattice parameters *a*_c_, *b*_c_, and *c*_c_ as a function of composition *x* at *T* = 12 K. The dashed vertical lines indicate the apparent morphotropic phase boundaries. Error bars on the lattice parameters are smaller than the data points and have been omitted for clarity.

To investigate the effect of halide substitution on the crystal structure and phase transitions, we carry out low-temperature XRD measurements on phase pure powder samples of the MAPb(Cl_*x*_Br_1–*x*_)_3_ series obtained by solid state synthesis over the full composition range (Figure 1, Table S1). We focus on this system because of its good miscibility over the entire composition range, unlike the closely related MAPb(Br_*x*_I_1–*x*_)_3_ system, which exhibits a wide miscibility gap at room temperature.^35^ Upon cooling, MAPbX_3_ (X = Cl, Br, and I) perovskites undergo symmetry-lowering displacive phase transitions constituting octahedral tilts and distortions, accompanied by successive order–disorder type restrictions on the orientational degrees of freedom of the MA cation.^37−40^ The phase transitions lower the unit cell symmetry from cubic through tetragonal to orthorhombic, which is manifested by successive splitting of the diffraction peaks as the temperature is lowered (Figure 1a,c).^39^ In contrast to the end members, we do not observe peak splitting in the mixed *x* = 0.5 composition (Figure 1b), indicating that the average structure retains its high-temperature cubic symmetry down to *T* = 12 K and suggests a suppression of the phase transitions. We observe an identical behavior in a wide composition range from *x* = 0.2 to *x* = 0.8 (Figure S1). We use the Le Bail refinement to extract the lattice parameters from the diffraction data using the TOPAS software^29,30^ (Figure S2). The obtained cubic (*Pm*3̅*m* space group) lattice parameters at *T* = 300 K as a function of composition show excellent agreement with Vegard’s law^36^ (Figure 1e). At temperatures below *T* = 300 K, we fit the diffraction data with the low symmetry orthorhombic *Pnma* space group to obtain pseudocubic lattice parameters (Figure 1d, Supporting Information section S2) as a function of temperature and composition. The pseudocubic lattice parameters at *T* = 12 K show apparent morphotropic phase boundaries (Figure 1f), highlighting the central compositional region where the high temperature cubic symmetry is retained. Starting on the Br-rich (left) side of the diagram (Figure 1f), we observe an orthorhombic splitting of the lattice parameters up to *x* = 0.10. At *x* = 0.15, parameters *a*_c_ and *b*_c_ coincide, indicating the existence of a tetragonal symmetry at this composition. From *x* = 0.2 until *x* = 0.8, the three lattice parameters are equal, implying the presence of a cubic symmetry for these compositions. On the Cl-rich (right) side, we observe a small orthorhombic splitting between *x* = 0.85 and *x* = 0.90, which increases as we approach the pure Cl end member.

### Phase Diagram of the MAPb(Cl_*x*_Br_1–*x*_)_3_ System

**Figure 2:**
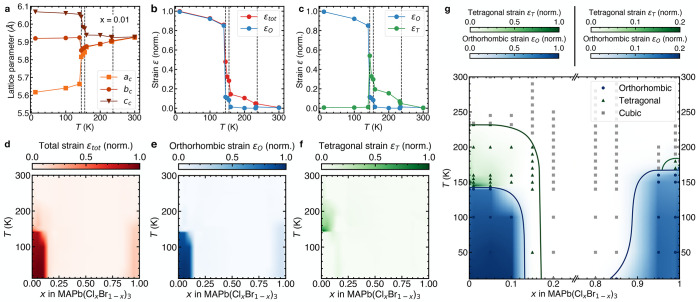
Macroscopic strain analysis and phase diagram of MAPb(Cl_*x*_Br_1–*x*_)_3_. (a–c) Representative results from strain analysis of variable temperature XRD measurements for MAPb(Cl_0.01_Br_0.99_)_3_. Vertical dashed lines represent phase transition temperatures of pure MAPbBr_3_.^37^ (a) Pseudocubic lattice parameters as a function of temperature for MAPb(Cl_0.01_Br_0.99_)_3_. (b) Normalized total strain ε_tot_ = *c*_c_/*a*_c_ – 1 and orthorhombic strain ε_O_ = *b*_c_/*a*_c_ – 1 as a function of temperature. (c) Normalized orthorhombic strain ε_O_ and tetragonal strain ε_T_ = ε_tot_ – ε_O_max(ε_tot_)/max(ε_O_) as a function of temperature. (d–f) Normalized and linearly interpolated ε_tot_, ε_O_ and ε_T_ strain maps for all measured compositions and temperatures. (g) Phase diagram of the MAPb(Cl_*x*_Br_1–*x*_)_3_ system obtained from overlaying the orthorhombic and tetragonal normalized strain maps and adding a linear transparency term to the color scale. Solid lines indicating phase boundaries are guides to the eye. Symbols denote individual XRD measurements and represent the corresponding crystal system assigned from the strain analysis. The color scale below *x* = 0.2 ranges from 0 (white) to 1 (color), and above *x* = 0.8 from 0 (white) to 0.2 (color) in order to clearly visualize both distortions of the Br- and Cl-rich compositions.

We use the macroscopic strain calculated from the pseudocubic lattice parameters as a proxy for the primary order parameter (the octahedral rotation angle)^41,42^ to construct a phase diagram of the MAPb(Cl_*x*_Br_1–*x*_)_3_ system (Figure 2). The tetragonal or orthorhombic character of the unit cell can be described by the deviation from the parent cubic structure (*a*_c_ = *b*_c_ = *c*_c_). The orthorhombic character can be described by the deviation between *a*_c_ and *b*_c_, which is given by the orthorhombic strain ε_O_
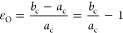


The tetragonal character is encompassed by the deviation between *a*_c_ and *c*_c_, which is given by the total strain ε_tot_ because it also encompasses a contribution from the orthorhombic strain
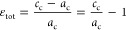


In order to separate the tetragonal strain ε_T_ from the orthorhombic strain ε_O_, we take the normalized difference between the total strain ε_tot_ and the orthorhombic strain ε_O_ as follows
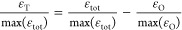
and after rearrangement
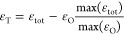


These parameters capture not only the strain magnitude but also the crystal symmetry without any prior assumptions on whether the system is cubic, tetragonal, or orthorhombic.

Starting with the pseudocubic lattice parameters of a representative composition (*x* = 0.01, Figure 2a), we calculate the orthorhombic and total strain (Figure 2b) and tetragonal strain (Figure 2c). Note that this analysis captures the sharp change in tetragonal strain parameter between *T* = 145 K and *T* = 155 K, which we attribute to the previously reported transition between the two distinct tetragonal phases (*I*4/*mcm* and *P*4/*mmm*) in the *x* = 0 end member.^37^ We repeat this analysis for the entire composition range (Figure S3), applying linear interpolation between the datapoints and normalize the data for each composition to the highest strain value (i.e., that of MAPbBr_3_ at *T* = 12 K) to obtain strain parameter maps (Figure 2d–f). These maps reveal the location of the cubic (white), tetragonal (green), and orthorhombic (blue) phase regions as a function of temperature and composition. We overlay the orthorhombic and tetragonal distortion maps to obtain the complete phase diagram of the MAPb(Cl_*x*_Br_1–*x*_)_3_ system (Figure 2g), which confirms the existence of sharp morphotropic phase boundaries at *x* = 0.15 (orthorhombic/tetragonal), *x* = 0.2 (tetragonal/cubic), and *x* = 0.85 (cubic/orthorhombic). The central region from *x* = 0.2 up to *x* = 0.85 is best described by a cubic symmetry down to *T* = 12 K. We hypothesize that the disorder introduced by halide substitution in the central region frustrates the long-range concerted octahedral rotations and distortions that constitute the phase transitions in the end members, leading to local uncorrelated distortions with an average cubic symmetry. Given the difference in size and electronegativity of the halides, this mechanism likely also involves a complex combination of local strain and frustrated interactions of the organic MA cations with their local anisotropic halide environment. Crucially, we note the similarity between the constituents and the phase diagram of MAPb(Cl_*x*_Br_1–*x*_)_3_ and those of orientational glass forming systems such as Na_1–*x*_K_*x*_CN, in which local random strains originating from alkali metal substitution frustrate the ordering of CN^–^ dipoles.^43,44^

### Local Strain in Mixed-Halide Hybrid Perovskites

**Figure 3:**
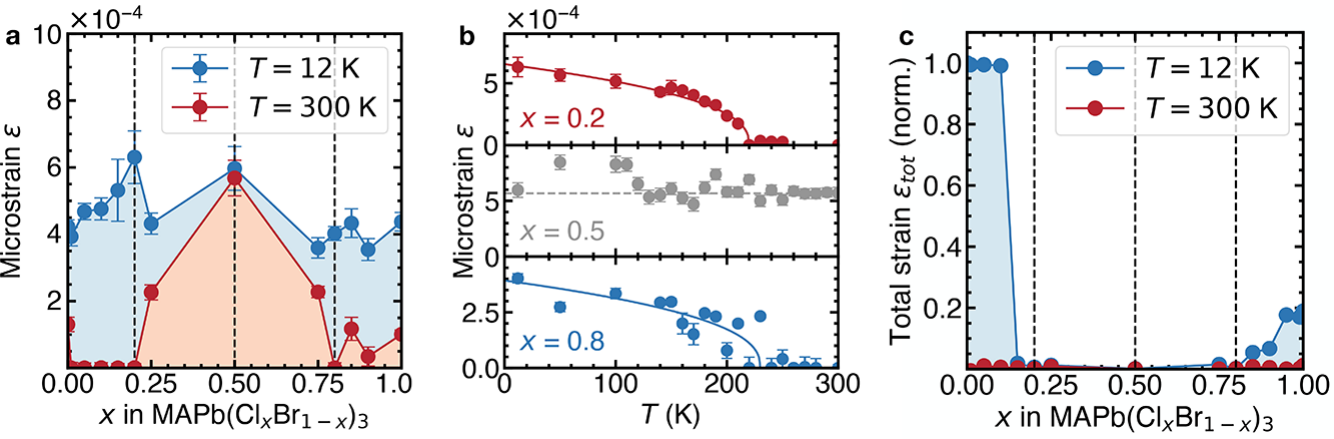
Microstrain analysis of MAPb(Cl_*x*_Br_1–*x*_)_3_. (a) Microstrain ε as a function of composition *x* obtained from XRD peak broadening at *T* = 12 K (blue) and *T* = 300 K (red). The compositions *x* = 0.2, *x* = 0.5 and *x* = 0.8 are depicted by dashed vertical lines. (b) Microstrain ε as a function of temperature for the three compositions depicted in (a). Solid lines for *x* = 0.2 and *x* = 0.8 are order parameter fits. The dashed line for *x* = 0.5 is a constant and serves as a guide to the eye. (c) Total strain parameter ε_tot_ as a function of composition at *T* = 12 K and *T* = 300 K obtained from the pseudocubic lattice parameters as shown in Figure 2. Dashed vertical lines denote the same compositions as in (a,b).

In order to verify the presence and role of local strains in the formation of the orientational glass, we extract the microstrain from the XRD peak broadening for each measurement in the MAPb(Cl_*x*_Br_1–*x*_)_3_ series (Figure 3) using TOPAS.^29^ For comparable results, the contribution of both size and microstrain is first refined for each composition at *T* = 300 K. For subsequent refinements at lower temperatures, we fix the size broadening term to the one obtained at *T* = 300 K and only let the microstrain broadening term refine (Figure 3a,b).

All compositions share a similar level of microstrain at *T* = 12 K (Figure 3a), whereas only compositions around *x* = 0.5 retain a significant amount of microstrain up to room temperature. The critical exponent behavior of the microstrain as a function of temperature for the compositions at the boundaries of the central strained region (Figure 3b, *x* = 0.2 and *x* = 0.8) can be attributed to the ferroelastic nature of MAPbX_3_ (X = Cl, Br, and I) perovskites,^45−47^ as the spontaneous microstrain and order parameter are strongly coupled in ferroelastic transitions.^48^ Order parameter fits of the form ε ∝ (*T*_c_ – *T*)^β^ for *x* = 0.2 and *x* = 0.8 yield a critical exponent of β = 0.4, which is in close agreement with the reported value of β = 0.42 for MAPbI_3_.^49^ The *x* = 0.5 composition retains a constant non-zero microstrain value throughout the measured temperature range. There is a clear correlation between the microstrain (Figure 3a) and the macroscopic strain (Figure 3c) for compositions close to the end members, while they are not correlated for the mixed compositions around *x* = 0.5. This corroborates the formation of an orientational glass in a broad range of mixed compositions, where local microstrains frustrate the collective long-range octahedral distortions, leading to suppression of the phase transitions and an average cubic structure without apparent macroscopic strains.

### Heat Capacity Measurements

**Figure 4:**
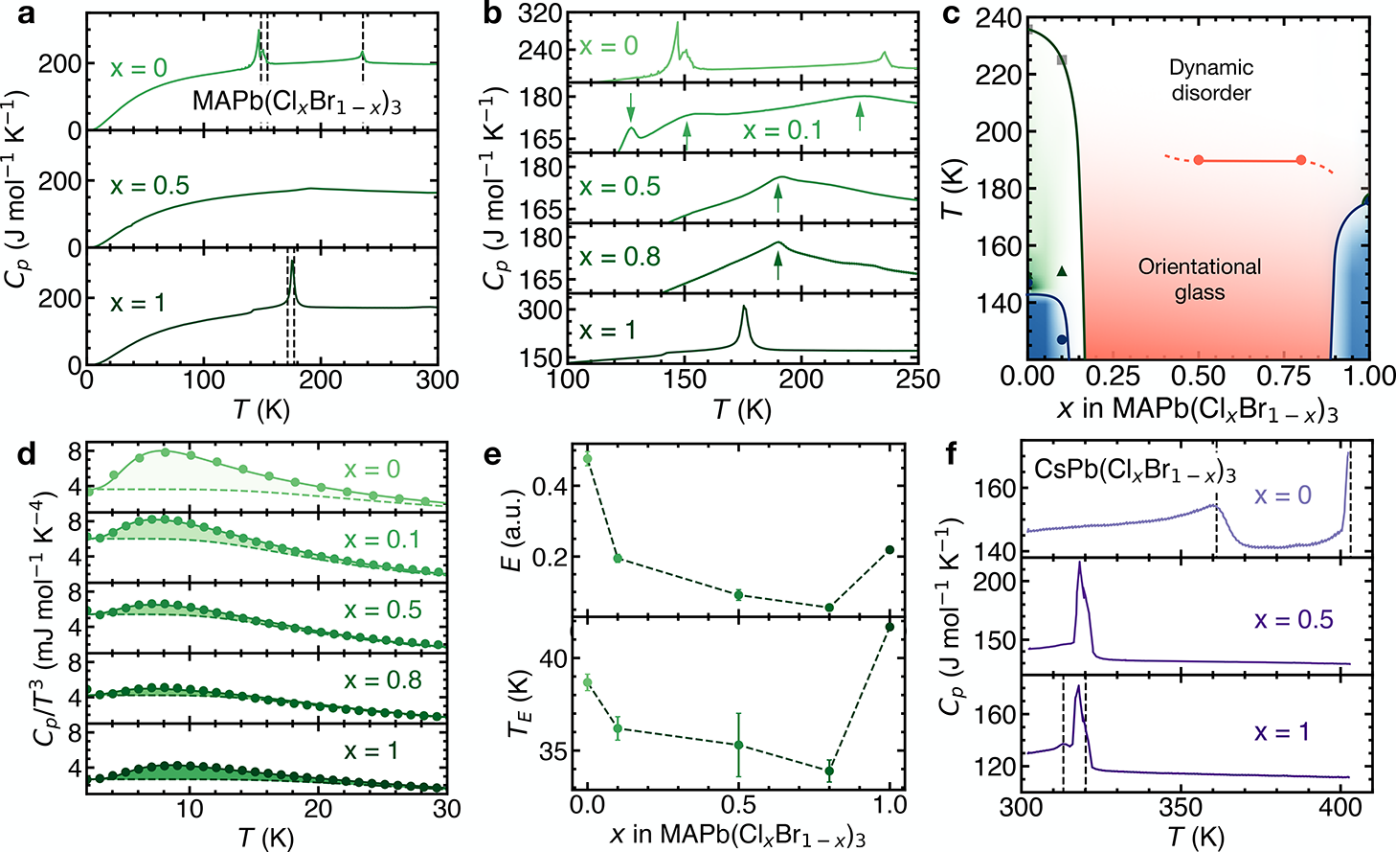
| Heat capacity measurements of MAPb(Cl_*x*_Br_1–*x*_)_3_ and CsPb(Cl_*x*_Br_1–*x*_)_3_. (a) Heat capacity of representative compositions as a function of temperature. Dashed vertical lines indicate the phase transition temperatures of the end members from the literature.^37^ (b) Close-up view of the phase transition temperature region of the measured compositions. Arrows indicate the maxima of the broad peaks observed in the mixed compositions. (c) Close-up region of the phase diagram with the peak positions from (b) plotted as a function of composition and temperature. White regions indicate cubic symmetry, green regions indicate tetragonal symmetry, blue regions indicate orthorhombic symmetry, and the red region illustrates the orientational glass. (d) View of the low-temperature region of *C*_p_/*T*^3^ for the different compositions of MAPb(Cl_*x*_Br_1–*x*_)_3_. Solid lines are fits and dashed lines represent the Debye contribution to the heat capacity (Supporting Information section S3). (e) Einstein contribution *E* (top) and Einstein temperature *T*_E_ (bottom) as a function of composition obtained from the fits in (d). (f) Heat capacity of the CsPb(Cl_*x*_Br_1–*x*_)_3_ system. Dashed vertical lines indicate the phase transition temperatures of the end members from the literature.^26,27^

To confirm the suppression of the phase transitions in MAPb(Cl_*x*_Br_1–*x*_)_3_ and characterize the orientational glass transition, we carry out heat capacity measurements as a function of temperature (Figure 4). The sharply diverging heat capacity at the phase transition temperatures in the end members is in good agreement with literature^37^ (Figure 4a). We note that the *x* = 0.5 composition lacks any sharp features in the heat capacity, indicating the absence of long-range collective reductions in the degrees of freedom of the MA cation and corroborating the suppression of the phase transitions indicated by the XRD data. However, we observe a broad feature around *T* = 190 K for the *x* = 0.5 composition. The broadening of heat capacity peaks in the presence of disorder is indicative of a shift from first-order to continuous character,^50^ which has been observed in related systems such as ferroic glasses,^51,52^ doped inorganic perovskites,^53^ and cation-substituted hybrid perovskites.^12,13,54^ The *x* = 0.1 composition shows three broad features that we attribute to the phase transition temperatures of the *x* = 0 end member shifted in temperature (Figure 4b). The broad features of the *x* = 0.5 and *x* = 0.8 compositions both peak around *T* = 190 K, which we interpret as the gradual freezing of the dynamically disordered organic cations, which results in an orientational glass at low temperatures. The heat capacity peak positions agree well with the diffraction data for the end members and reveal a broad glass transition region in the center of the phase diagram (Figure 4c).

In order to elucidate the role of the organic MA cation in the orientational glass state, we examine the low-temperature region of *C*_p_/*T*^3^ (Figure 4d). We observe a broad feature around *T* = 8 K for all compositions, indicating a deviation from the ideal *C*_p_ ∝ *T*^3^ Debye behavior, which has been attributed to a low-energy Einstein oscillator in the form of a rattling rigid body or atomic species in hybrid perovskites^54^ and other framework materials.^55^ By fitting the data with a simple model (Supporting Information section S3), we extract the Einstein contribution *E* and its characteristic temperature *T*_E_ as a function of composition (Figure 4e). We note the existence of a minimum in the Einstein contribution *E* at *x* = 0.5, superimposed on a linearly decreasing trend with increasing *x*. We interpret the linear trend as a gradual suppression of the low-temperature MA cation dynamics as a result of stronger hydrogen bonding interactions, due to the decrease in unit cell volume with increasing *x* at low temperatures (Figure S4). We ascribe the minimum in the Einstein contribution to the disorder in the local halide environment, which is the largest at *x* = 0.5. In these highly disordered compositions, we expect stronger hydrogen bonding interactions of the MA cation with the more electronegative Cl^–^, resulting in preferential orientations and a concomitant decrease in MA cation motion. The Einstein temperature *T*_E_ varies similarly with *x* as the Einstein contribution (Figure 4e, bottom). The vibrational frequency of 25 cm^–1^ associated with the average Einstein temperature (*T*_E_ = 37 K) is in reasonable agreement with the calculated low energy phonon mode in MAPbBr_3_ at 29.4 cm^–1^, involving concerted octahedral rotations and MA translations.^56^ This supports the hypothesis that the Einstein oscillator is likely the rattling MA cation and that its dynamics are slowed in the orientational glass state of the mixed compositions.

In order to confirm the key role of the organic MA cation in the suppression of phase transitions, we repeat the heat capacity measurements with the closely related inorganic CsPb(Cl_*x*_Br_1–*x*_)_3_ system (Figure 4f). This system undergoes the same sequence of symmetry lowering phase transitions as the hybrid system, with the transitions shifted to higher temperatures. The phase transition temperatures and heat capacity features of the end members agree well with the previous literature reports.^26,27^ In stark contrast to MAPb(Cl_*x*_Br_1–*x*_)_3_, the *x* = 0.5 composition exhibits similar heat capacity features as the CsPbCl_3_ end member. Moreover, the room-temperature crystal structure of the *x* = 0.5 composition is best described by the same orthorhombic (*Pnma*) space group as the end member compositions (Figures S5, S6), providing strong evidence that the phase transitions are not suppressed in the inorganic mixed-halide system.

### DFT Calculations

**Figure 5:**
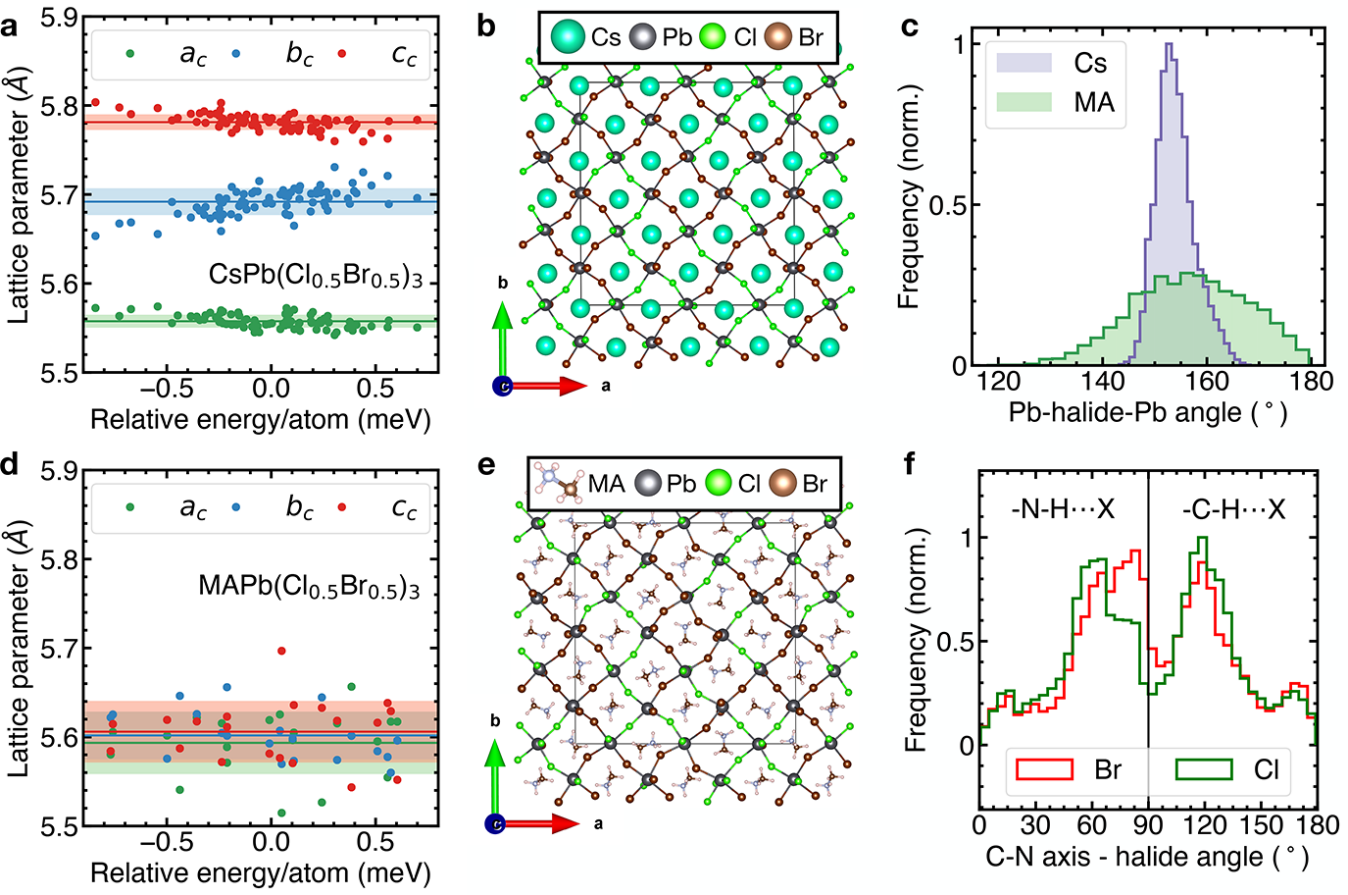
DFT calculations of MAPb(Cl_*x*_Br_1–*x*_)_3_ and CsPb(Cl_*x*_Br_1–*x*_)_3_. Pseudocubic lattice parameters extracted from simulated CsPb(Cl_0.5_Br_0.5_)_3_ (a) and MAPb(Cl_0.5_Br_0.5_)_3_ (d) supercells as a function of the relative energy per atom. Solid lines are mean values for each lattice parameter, shaded regions indicate one standard deviation. Ball and stick model of one layer of the CsPb(Cl_0.5_Br_0.5_)_3_ (b) and MAPb(Cl_0.5_Br_0.5_)_3_ (e) supercells with the same halide distribution. (c) Normalized distribution of Pb–halide–Pb angles for CsPb(Cl_0.5_Br_0.5_)_3_ (purple) and MAPb(Cl_0.5_Br_0.5_)_3_ (green). (f) Normalized distribution of angles between the C–N axis of the MA cation and the lines connecting its center of mass to the nearest neighbor halides (denoted by X) shown in green (X = Cl) and red (X = Br). Angles below 90° correspond to the case where the N-side of the MA molecule is pointing toward the halide (denoted by −N–H···X), angles above 90° correspond to the case where the C-side is pointing toward the halide (denoted by −C–H···X).

To investigate the influence of the cation species (Cs and MA) on the structural properties of the mixed-halide perovskites, we perform DFT calculations using the Vienna Ab Initio Simulation Package (VASP, v5.4).^57,58^ We first create 78 supercells (3 × 2 × 3) of orthorhombic (*Pnma*) CsPb(Cl_0.5_Br_0.5_)_3_ with the Cl and Br atoms randomly distributed across the halide sites. We let each of the supercells relax fully, resulting in static DFT structures that approximate the *T* = 0 K experimental structures. The relaxed structures show three distinct lattice parameters (Figure 5a), indicating an orthorhombic symmetry and no suppression of the phase transitions, consistent with the results from our XRD and heat capacity measurements.

We use the four halide distributions with the lowest energy CsPb(Cl_0.5_Br_0.5_)_3_ perovskite structures to construct the supercells for the hybrid perovskites. For each halide distribution, we create five MAPb(Cl_0.5_Br_0.5_)_3_ structures with randomly oriented MA molecules. This approach captures both the disorder introduced by the halide mixing, as well as the rotational degrees of freedom of the MA molecules using a computationally manageable set of structures. The resulting 20 structures are then relaxed, during which we observe an increase of *a*_c_ and a decrease of *b*_c_ and *c*_c_, causing them to overlap within one standard deviation (Figure 5d). This indicates that MAPb(Cl_0.5_Br_0.5_)_3_, contrary to CsPb(Cl_0.5_Br_0.5_)_3_, energetically favors an average cubic crystal structure, consistent with the suppression of the phase transitions observed in our XRD and heat capacity measurements. This is further exemplified by the absence of long-range concerted octahedral tilts and distortions in the MAPb(Cl_0.5_Br_0.5_)_3_ supercell (Figure 5e), which are present in the CsPb(Cl_0.5_Br_0.5_)_3_ supercell (Figure 5b). We find a broader Pb–halide–Pb angle distribution in MAPb(Cl_0.5_Br_0.5_)_3_ that is shifted toward the ideal cubic value of 180°, compared to the sharp distribution in CsPb(Cl_0.5_Br_0.5_)_3_ (Figure 5c). Further, we uncover an asymmetry in the orientation preferences of the MA cations with respect to their halide environment (Figures 5f, S7). The peaks at 60 and 120° correspond to orientations of the C–N axis that align the N–H and C–H axes toward the halides, maximizing the hydrogen bonding interaction. We observe a narrower angular distribution for the N–H···Cl interaction compared to the N–H···Br interaction (Figure 5f, C–N axis—halide angle < 90°), indicating the preferential alignment of the MA cations to facilitate the stronger N–H···Cl bond. We do not observe the same angular asymmetry when considering the weaker C–H···X bond (Figure 5f, C–N axis—halide angle > 90°), but the angular distribution does show a higher number of MA cations oriented toward the more electronegative Cl^–^ than toward Br^–^, again suggesting preferential alignment.

### Low-Temperature PL

**Figure 6:**
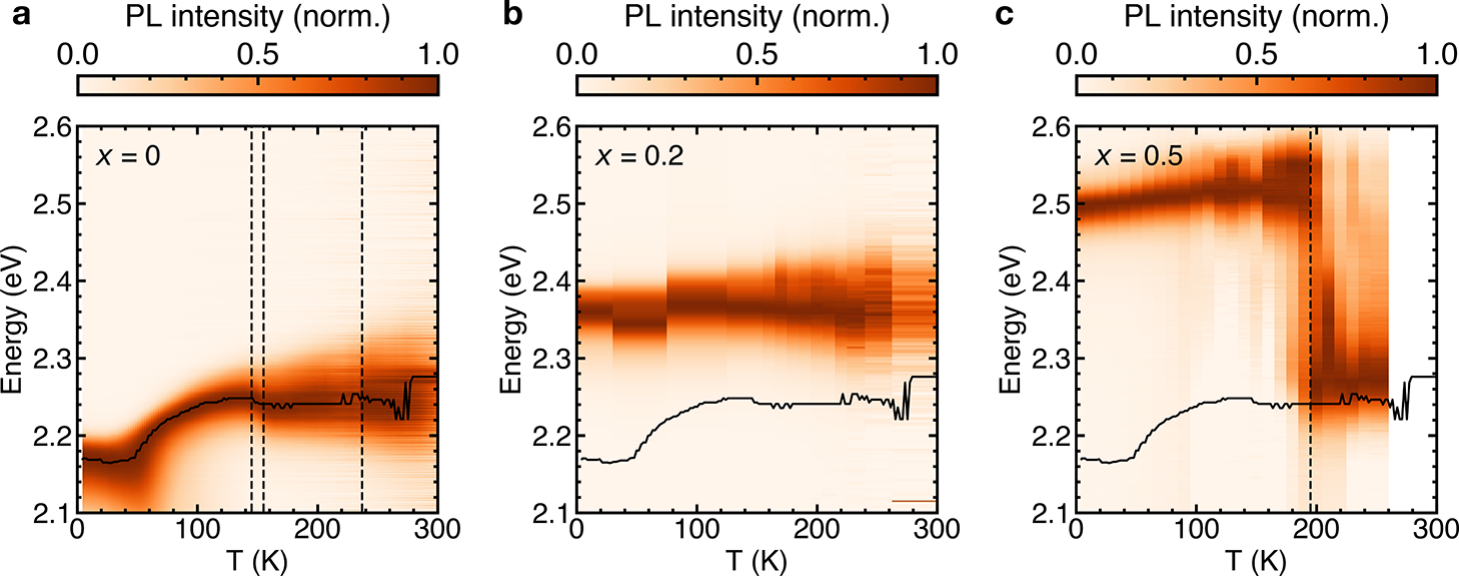
Low-temperature PL of MAPb(Cl_*x*_Br_1–*x*_)_3_. Normalized PL as a function of temperature for the *x* = 0 (a), *x* = 0.2 (b) and *x* = 0.5 (c) compositions. The black vertical dashed lines in (a) represent the phase transition temperatures of *x* = 0 from literature,^37^ and the onset of photo-induced halide segregation in (c). The black solid lines trace the PL peak energy of the *x* = 0 composition for comparison.

To assess the impact of the structure on the optoelectronic properties of MAPb(Cl_*x*_Br_1–*x*_)_3_, we perform low-temperature PL measurements (Figure 6). For the *x* = 0 composition, we find the previously reported PL emission features^59^ (Figures 6a, S8a). With decreasing temperature from *T* = 300 K, the PL spectra narrow and start to redshift from *T* = 150 K, with additional anomalous broadening (Supporting Information section S4, Figure S8a), likely connected to the low-energy rattling of the MA cation.^54^ We observe no anomalous broadening of the PL for the *x* = 0.2 and *x* = 0.5 compositions (Figure S8b,c), in agreement with a reduction in MA cation motion as a result of preferential alignment and a smaller unit cell volume compared to *x* = 0. In contrast to *x* = 0, the *x* = 0.5 composition shows a sharp jump in the spectral peak position around *T* = 180 K (Figure 6c), which can be attributed to emission from Br-rich regions following photo-induced halide segregation.^16,24,60−62^ This abrupt change in the PL spectrum for *x* = 0.5 occurs in the vicinity of the orientational glass transition temperature, which indicates a link between the glassy structure and optoelectronic properties of the material. These observations show that the microstrain in the orientational glass state of the *x* = 0.5 mixed-halide composition is strongly correlated to the halide segregation. To confirm this interpretation, we perform additional PL emission experiments on the *x* = 0.2 composition (Figure 6b), the composition closest to *x* = 0.5 without any residual microstrain at room temperature (Figure 3b). We find no sharp jumps in the PL spectrum under illumination, in stark contrast to the *x* = 0.5 case, confirming the formation of a photostable material system once the microstrain is removed.

## Conclusions

Our results reveal the presence of a disorder- and frustration-driven orientational glass with associated microstrains that are strongly correlated with photo-induced halide segregation in mixed-halide hybrid perovskites. The orientational glass is formed as a result of local preferential alignment of the MA cations due to their surrounding disordered halide environment. The incompatibility of neighboring MA orientations combined with microstrains from the differently sized halide ions frustrate the long-range concerted octahedral tilting, resulting in a suppression of the phase transitions. The residual static microstrains above the orientational glass transition temperature likely provide the conditions necessary for halide diffusion, assisted by transient strain gradients from polarons.^23,63^ Our findings explain the unexpected photostability of certain compositions of mixed-halide hybrid perovskites,^64^ namely, the ones without residual microstrain at room temperature. It also rationalizes the substitution of MA with other A-site cations to increase the photostability by relieving microstrain from frustrated regions. Our results demonstrate the need for novel strain management approaches to achieve photostable hybrid metal–halide perovskites, through rational selection of additives, substrates, and fabrication methods.^65^

## Supporting Information Available

Table of measured compositions and temperatures for MAPb(Cl_*x*_Br_1–*x*_)_3_, temperature and composition-dependent XRD data for MAPb(Cl_*x*_Br_1–*x*_)_3_, Le Bail refinements of the XRD data for MAPb(Cl_*x*_Br_1–*x*_)_3_, strain analysis of the XRD data as a function of temperature and composition for MAPb(Cl_*x*_Br_1–*x*_)_3_, definition of the pseudocubic lattice parameters for MAPb(Cl_*x*_Br_1–*x*_)_3_, low-temperature heat capacity model for MAPb(Cl_*x*_Br_1–*x*_)_3_, unit cell volume as a function of composition for MAPb(Cl_*x*_Br_1–*x*_)_3_, Rietveld refinements of XRD data for CsPb(Cl_*x*_Br_1–*x*_)_3_, and illustration of the DFT analysis PL linewidth analysis (PDF)
