# Supplementary material for: Impact of Orientational Glass Formation and Local Strain on Photo-Induced Halide Segregation in Hybrid Metal-Halide Perovskites
Source: J Phys Chem C Nanomater Interfaces. 2021 Jun 30;125(27):15025–34. doi: 10.1021/acs.jpcc.1c03169 (PMC8287560; doi:10.1021/acs.jpcc.1c03169)
Supplement: Supplementary file 1 — jp1c03169_si_001.pdf [file jp1c03169_si_001.pdf]

Supplementary materials for

Impact of Orientational Glass Formation and Local Strain on Photo-induced Halide Segregation in Hybrid Metal-halide Perovskites

Tim W.J. van de Goor[1], Yun Liu[1], Sascha Feldmann[1], Sean A. Bourelle[1], Timo Neumann[1,2], Thomas Winkler[1,3], Nicola D. Kelly[1], Cheng Liu[1], Michael A. Jones[4], Steffen P. Emge[4], Richard H. Friend[1], Bartomeu Monserrat[1,5], Felix Deschler[1,2,†], Siân E. Dutton[1,*]

[1]Cavendish Laboratory, University of Cambridge, J. J. Thomson Avenue, Cambridge, CB3 0HE, UK

[2]Walter Schottky Institut and Physik Department, Technische Universität München, Am Coulombwall 4, 85748 Garching, Germany

[3]Department of Physics and Astronomy, Aarhus University, 8000 Aarhus C, Denmark

[4]Department of Chemistry, University of Cambridge, Lensfield Road, Cambridge, CB2 1EW, UK

[5]Department of Materials Science and Metallurgy, University of Cambridge, 27 Charles Babbage Road, Cambridge, CB3 0FS, UK

†E-mail: Felix.Deschler@wsi.tum.de

*E-mail: sed33@cam.ac.uk

Table S1 | Summary of compositions and temperatures measured of the $MAPb(Cl_xBr_{1-x})_3$ system using temperature dependent X-ray diffraction.

**Composition *x***	**Temperatures (K)**
0	12.0, 140.0, 148.0, 150.0, 153.0, 195.0, 225.0, 238.0, 245.0, 300.0
0.01	12.0, 100.0, 140.0, 145.0, 150.0, 155.0, 160.0, 200.0, 232.0, 233.0, 234.0, 300.0
0.05	12.0, 50.0, 100.0, 140.0, 145.0, 150.0, 155.0, 160.0, 200.0, 232.0, 234.0, 245.0, 300.0
0.1	12.0, 100.0, 150.0, 160.0, 200.0, 232.0, 245.0, 300.0
0.15	12.0, 50.0, 140.0, 150.0, 160.0, 170.0, 180.0, 190.0, 200.0, 220.0, 230.0, 240.0, 250.0, 260.0, 270.0, 280.0, 290.0, 300.0
0.2	12.0, 50.0, 100.0, 140.0, 150.0, 160.0, 170.0, 180.0, 190.0, 200.0, 210.0, 220.0, 230.0, 240.0, 250.0, 300.0
0.25	12.0, 25.0, 50.0, 75.0, 100.0, 125.0, 140.0, 150.0, 155.0, 160.0, 300.0
0.5	12.0, 50.0, 100.0, 110.0, 120.0, 130.0, 140.0, 150.0, 160.0, 170.0, 180.0, 190.0, 200.0, 210.0, 220.0, 230.0, 240.0, 250.0, 260.0, 270.0, 280.0, 290.0, 300.0
0.75	12.0, 300.0
0.8	12.0, 50.0, 100.0, 140.0, 150.0, 160.0, 170.0, 180.0, 190.0, 200.0, 210.0, 220.0, 230.0, 240.0, 250.0, 260.0, 270.0, 280.0, 300.0
0.85	12.0, 50.0, 100.0, 140.0, 150.0, 160.0, 170.0, 180.0, 190.0, 200.0, 210.0, 220.0, 230.0, 240.0, 250.0, 260.0, 270.0, 280.0, 290.0, 300.0
0.9	12.0, 300.0
0.95	12.0, 50.0, 100.0, 150.0, 160.0, 170.0, 180.0, 190.0, 200.0, 300.0
0.99	12.0, 50.0, 100.0, 150.0, 160.0, 170.0, 180.0, 190.0, 200.0, 210.0, 220.0, 230.0, 300.0
1	12.0, 50.0, 100.0, 150.0, 170.0, 175.0, 180.0, 200.0, 250.0, 300.0

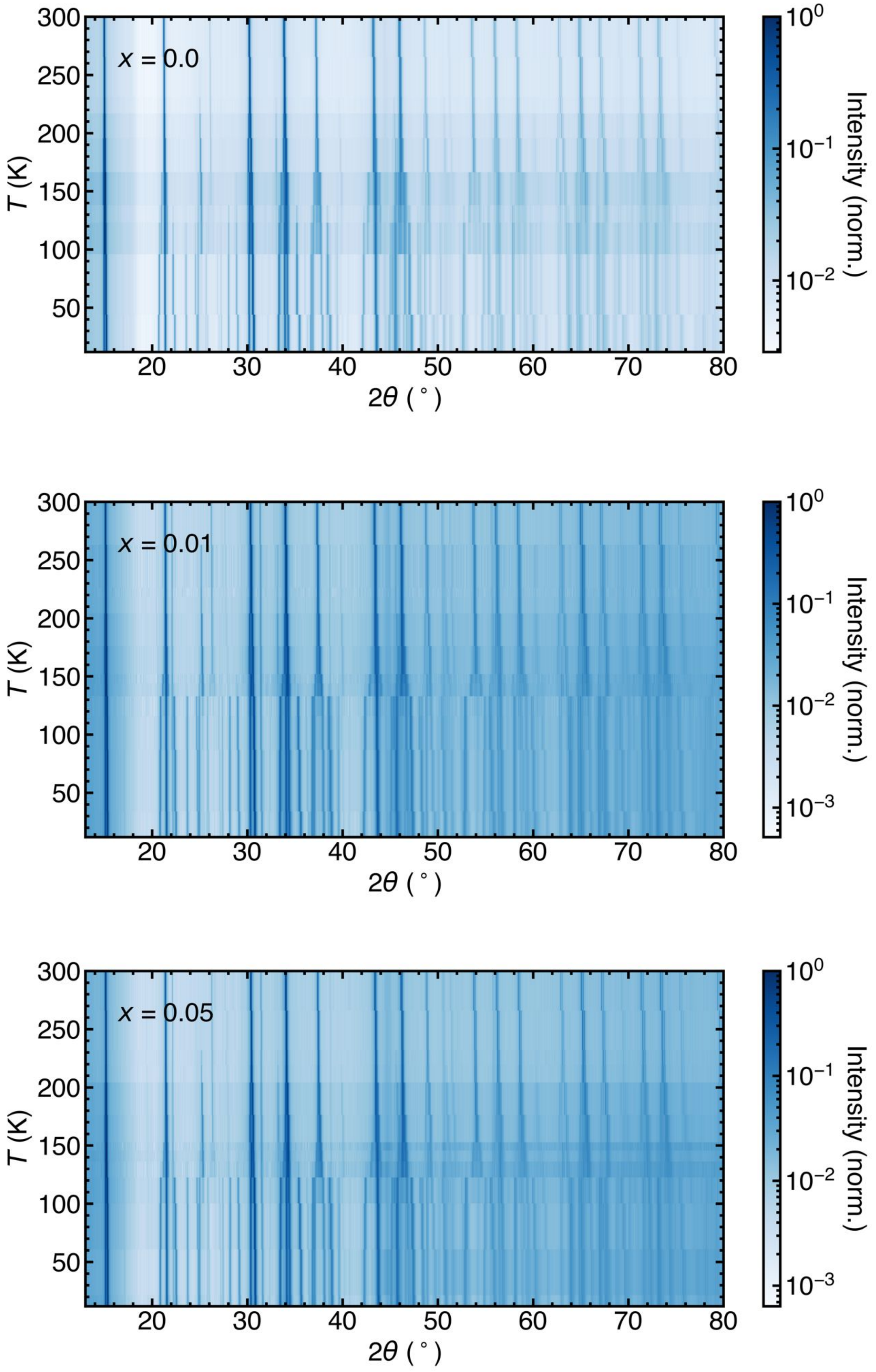

x = 0.0
T (K)
2θ (°)
Intensity (norm.)
x = 0.01
T (K)
2θ (°)
Intensity (norm.)
x = 0.05
T (K)
2θ (°)
Intensity (norm.)

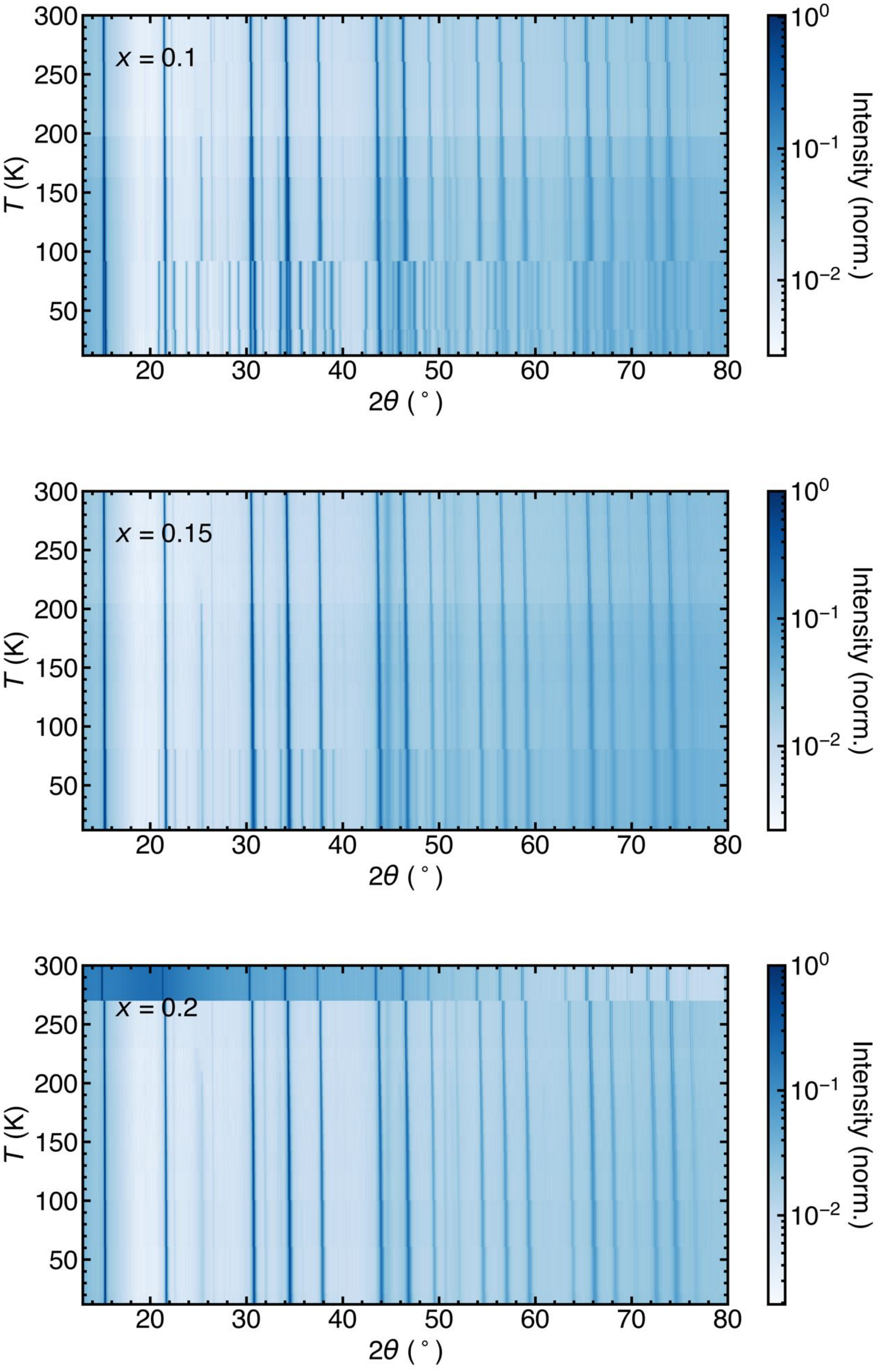
x = 0.1
x = 0.15
x = 0.2
T (K)
2θ (°)
Intensity (norm.)
10^0
10^-1
10^-2
300
250
200
150
100
50
20
30
40
50
60
70
80

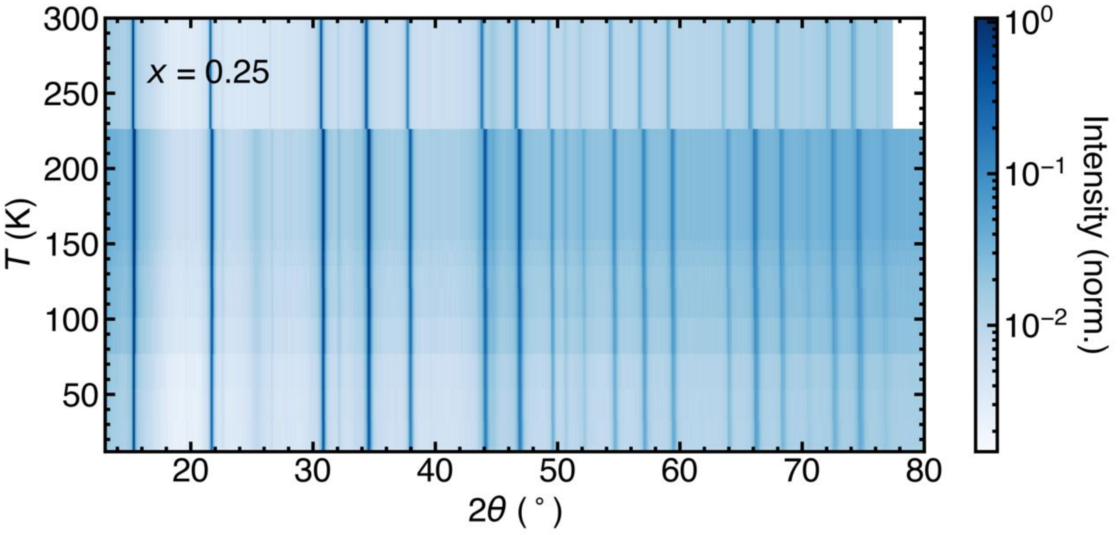
x = 0.25
T (K)
2θ (°)
Intensity (norm.)

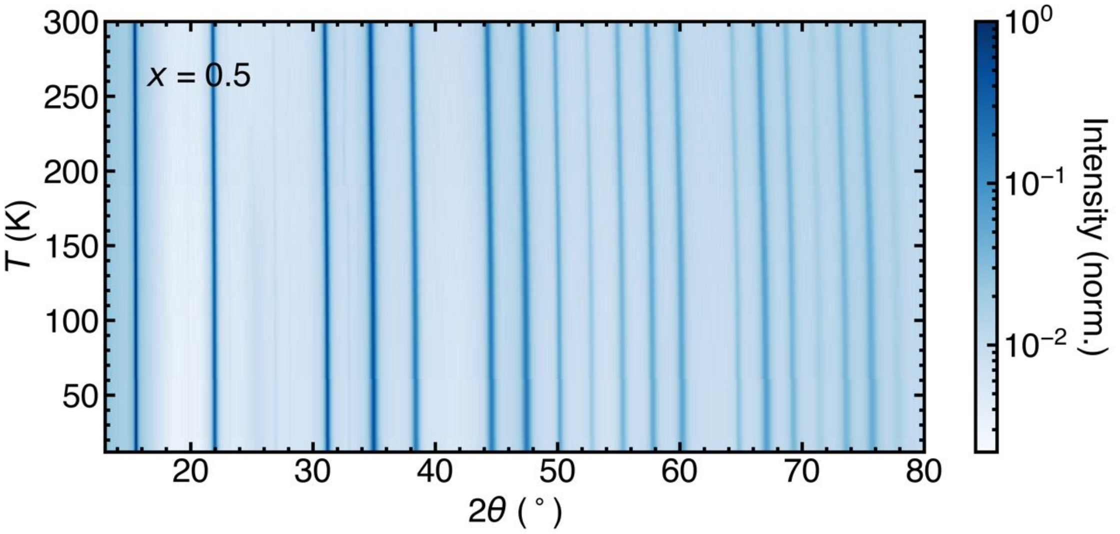
x = 0.5
T (K)
2θ (°)
Intensity (norm.)

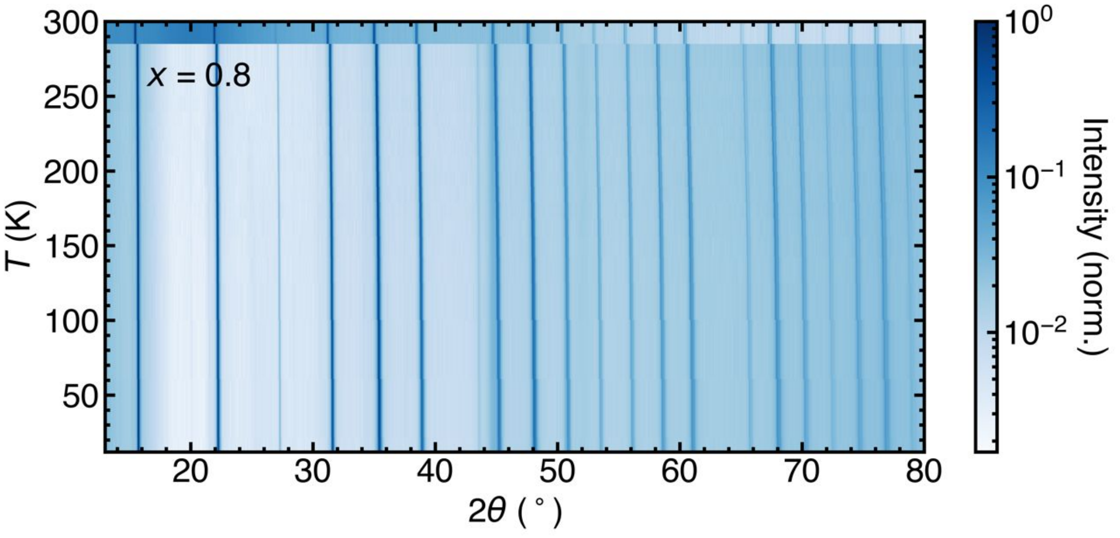
x = 0.8
T (K)
2θ (°)
Intensity (norm.)

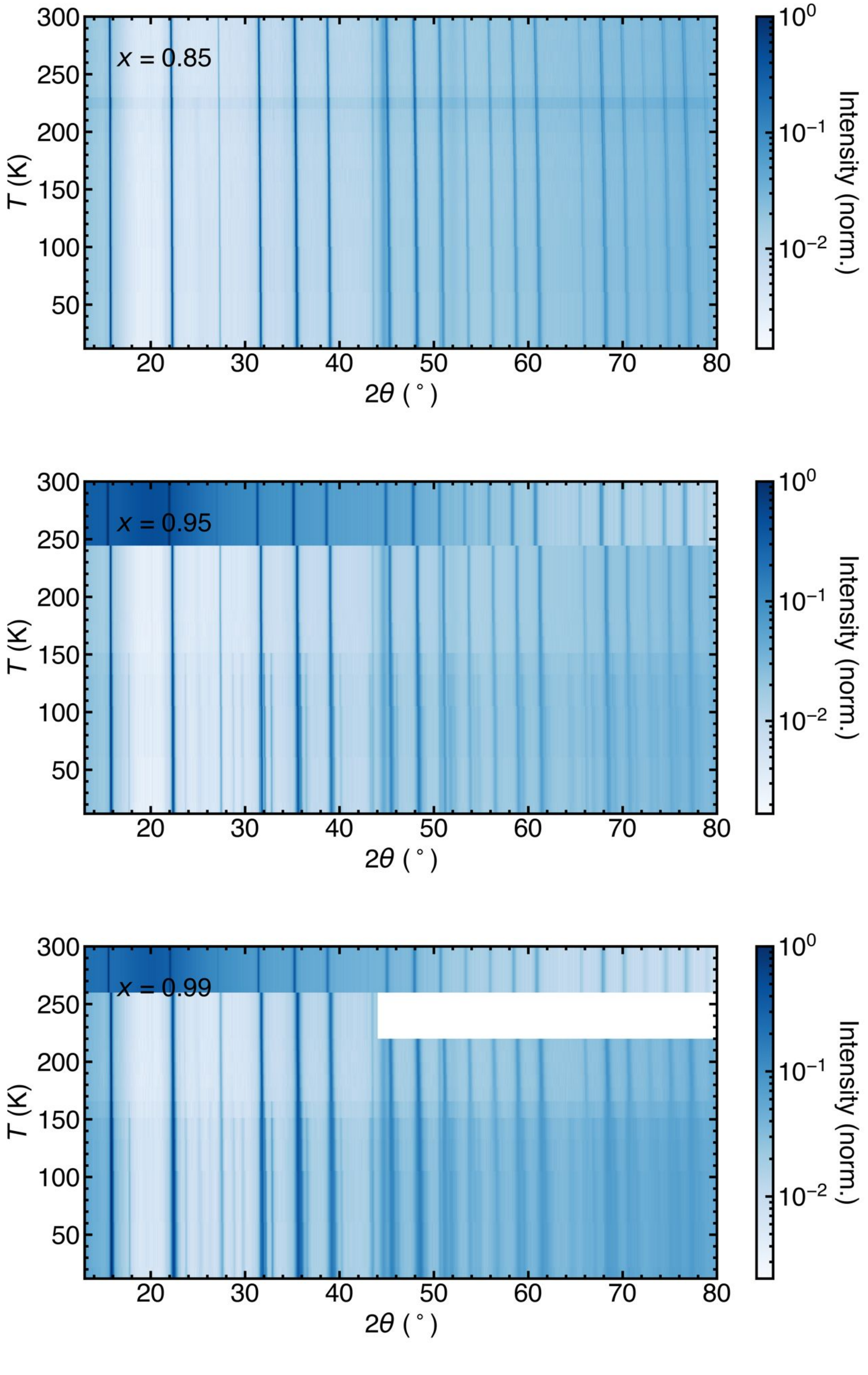

x = 0.85
x = 0.95
x = 0.99
T (K)
2θ (°)
Intensity (norm.)
300
250
200
150
100
50
20
30
40
50
60
70
80
10^0
10^−1
10^−2

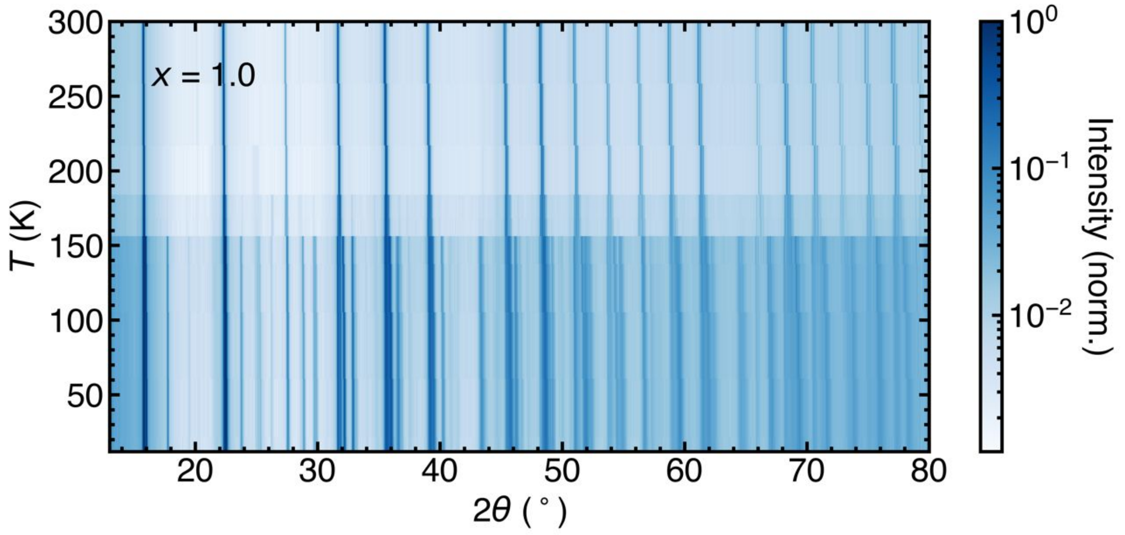

Figure S1 | Low temperature X-ray diffraction data of $MAPb(Cl_xBr_{1-x})_3$ showing normalised intensity as a function of the diffraction angle 2θ and temperature over the measured composition range. All data was collected using the Oxford Cryosystems PheniX, except for the room temperature data collected from the *x* = 0.2, 0.8, 0.95, and 0.99 samples. This was collected using a polymer inert atmosphere holder in the regular configuration of the diffractometer, causing a broad background feature around 2θ = 20°.

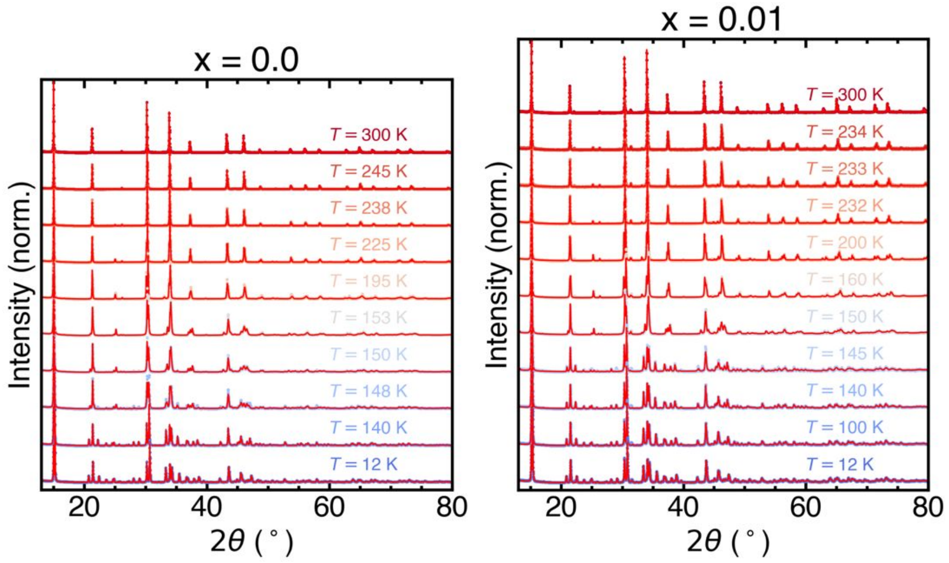

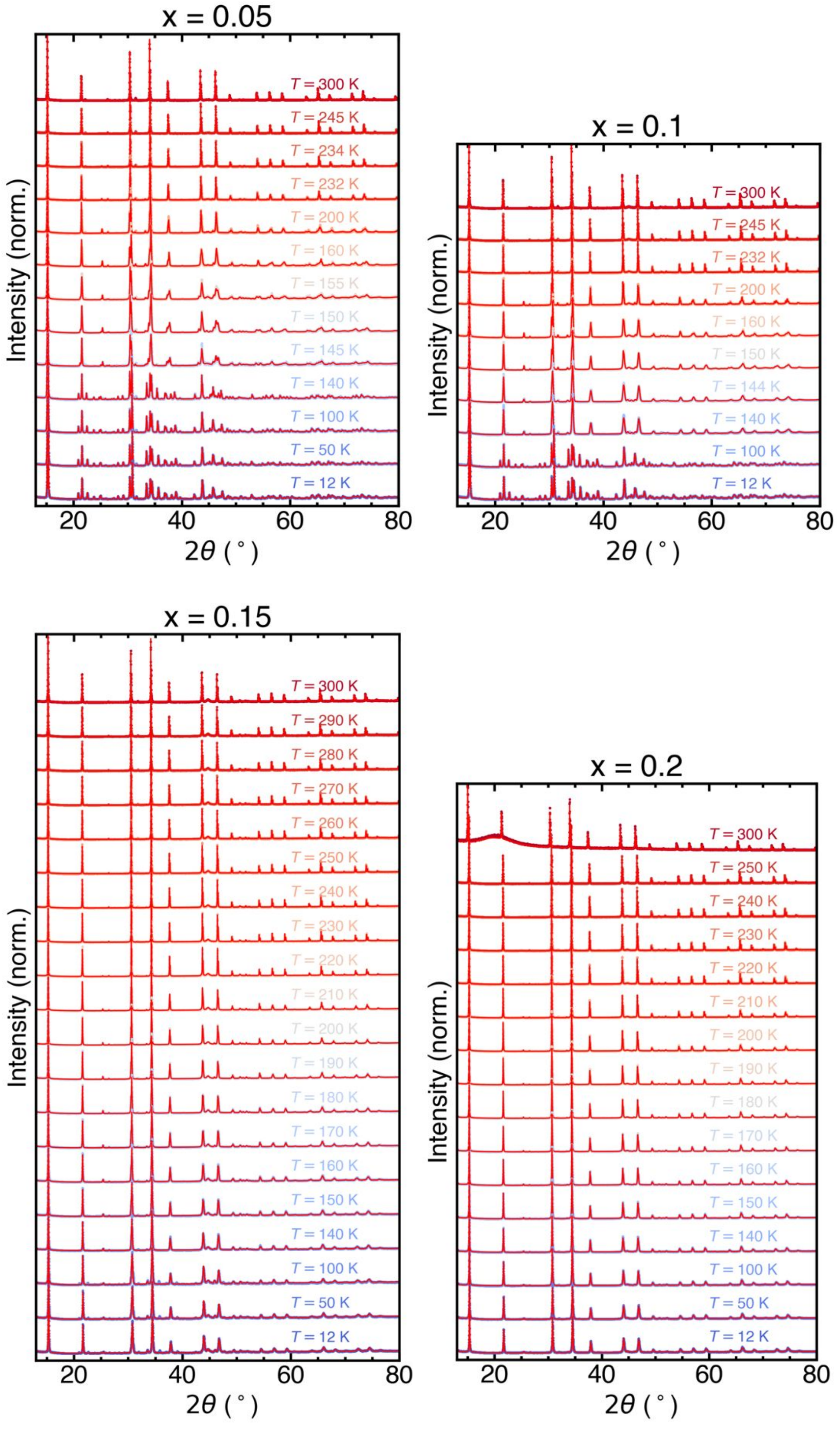
x = 0.05
T = 300 K
T = 245 K
T = 234 K
T = 232 K
T = 200 K
T = 160 K
T = 155 K
T = 150 K
T = 145 K
T = 140 K
T = 100 K
T = 50 K
T = 12 K
Intensity (norm.)
2θ (°)
x = 0.1
T = 300 K
T = 245 K
T = 232 K
T = 200 K
T = 160 K
T = 150 K
T = 144 K
T = 140 K
T = 100 K
T = 12 K
Intensity (norm.)
2θ (°)
x = 0.15
T = 300 K
T = 290 K
T = 280 K
T = 270 K
T = 260 K
T = 250 K
T = 240 K
T = 230 K
T = 220 K
T = 210 K
T = 200 K
T = 190 K
T = 180 K
T = 170 K
T = 160 K
T = 150 K
T = 140 K
T = 100 K
T = 50 K
T = 12 K
Intensity (norm.)
2θ (°)
x = 0.2
T = 300 K
T = 250 K
T = 240 K
T = 230 K
T = 220 K
T = 210 K
T = 200 K
T = 190 K
T = 180 K
T = 170 K
T = 160 K
T = 150 K
T = 140 K
T = 100 K
T = 50 K
T = 12 K
Intensity (norm.)
2θ (°)

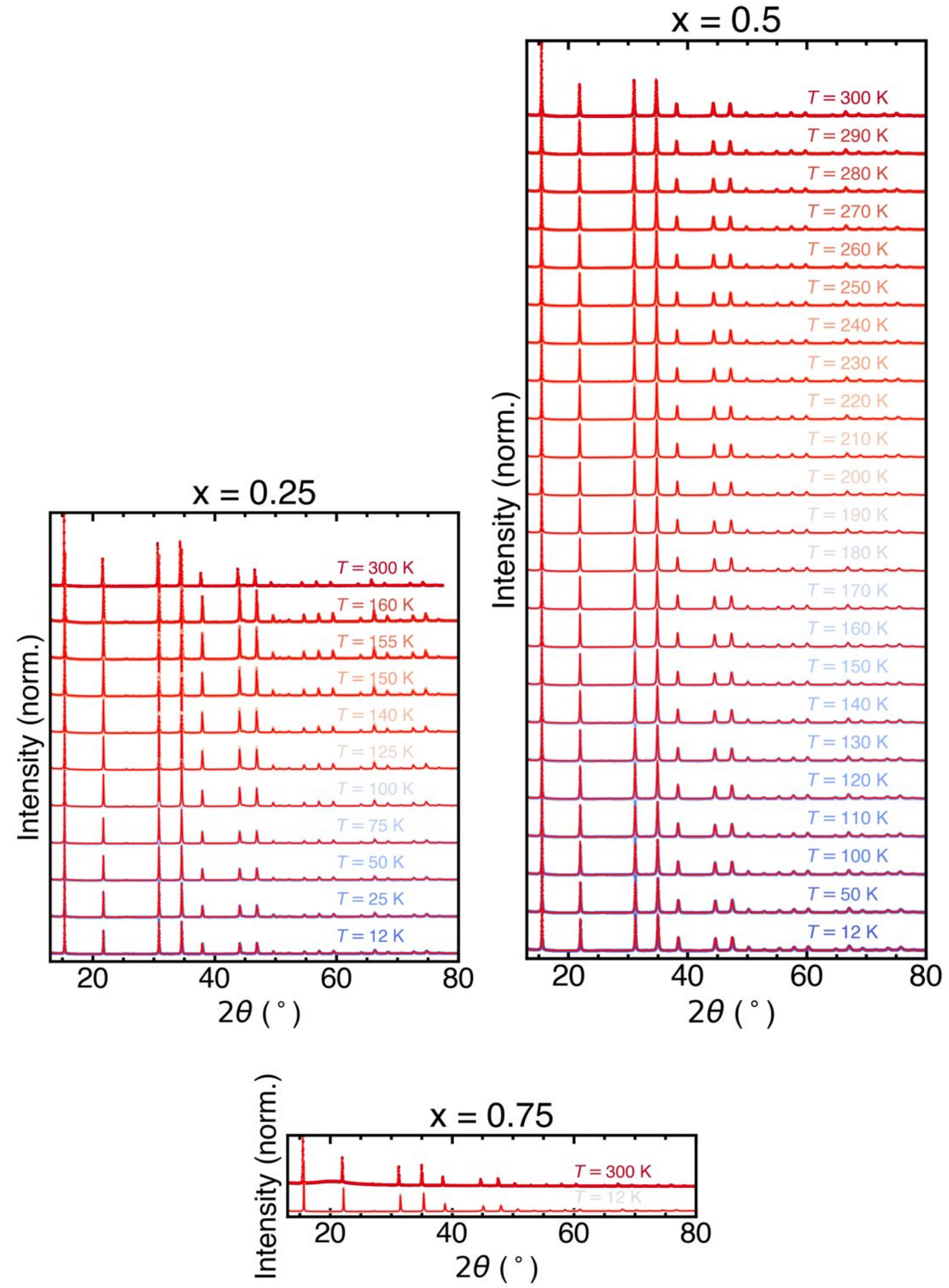
x = 0.5
T = 300 K
T = 290 K
T = 280 K
T = 270 K
T = 260 K
T = 250 K
T = 240 K
T = 230 K
T = 220 K
T = 210 K
T = 200 K
T = 190 K
T = 180 K
T = 170 K
T = 160 K
T = 150 K
T = 140 K
T = 130 K
T = 120 K
T = 110 K
T = 100 K
T = 50 K
T = 12 K
Intensity (norm.)
2θ (°)
x = 0.25
T = 300 K
T = 160 K
T = 155 K
T = 150 K
T = 140 K
T = 125 K
T = 100 K
T = 75 K
T = 50 K
T = 25 K
T = 12 K
Intensity (norm.)
2θ (°)
x = 0.75
T = 300 K
T = 12 K
Intensity (norm.)
2θ (°)

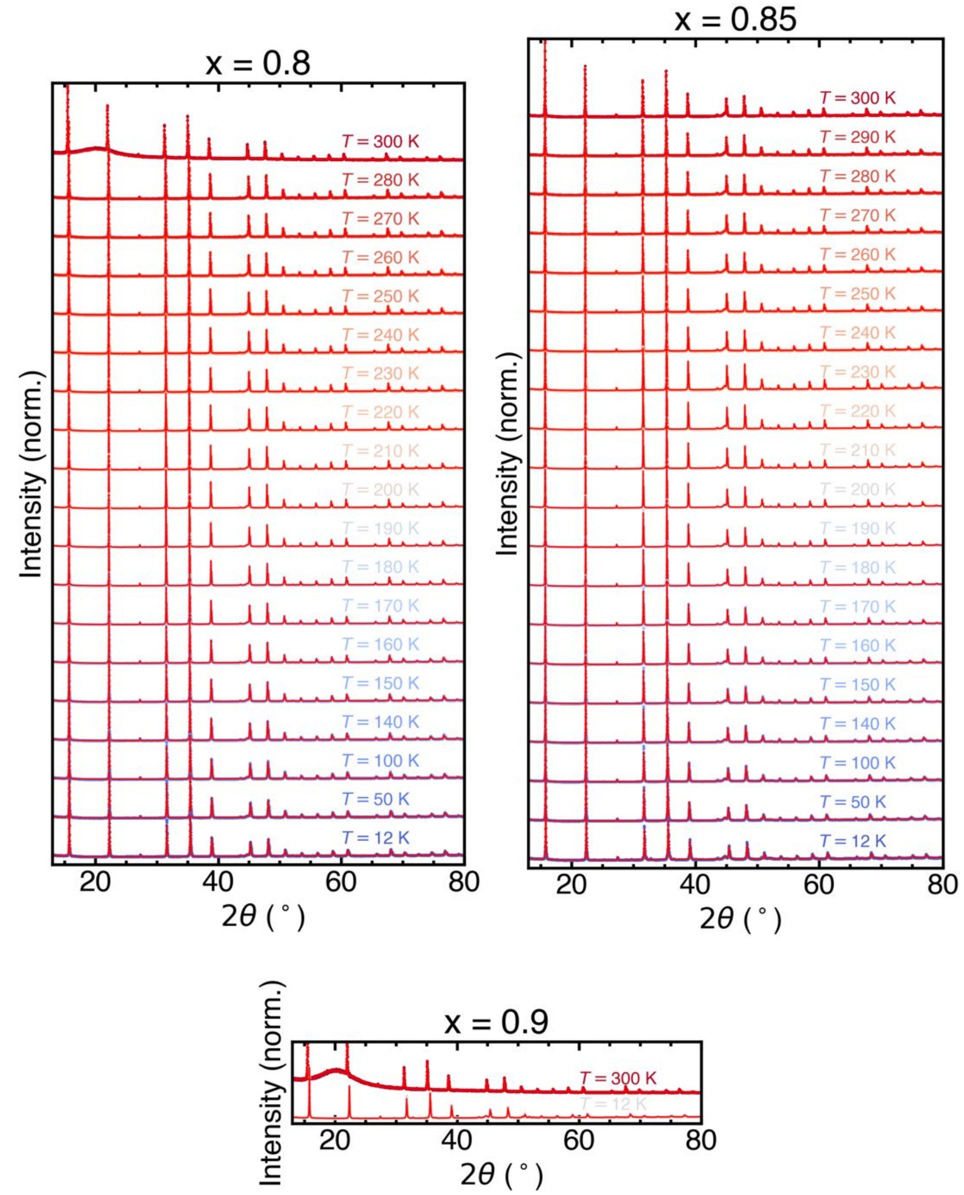
x = 0.8
x = 0.85
x = 0.9
Intensity (norm.)
2θ (°)
T = 300 K
T = 290 K
T = 280 K
T = 270 K
T = 260 K
T = 250 K
T = 240 K
T = 230 K
T = 220 K
T = 210 K
T = 200 K
T = 190 K
T = 180 K
T = 170 K
T = 160 K
T = 150 K
T = 140 K
T = 100 K
T = 50 K
T = 12 K
20
40
60
80

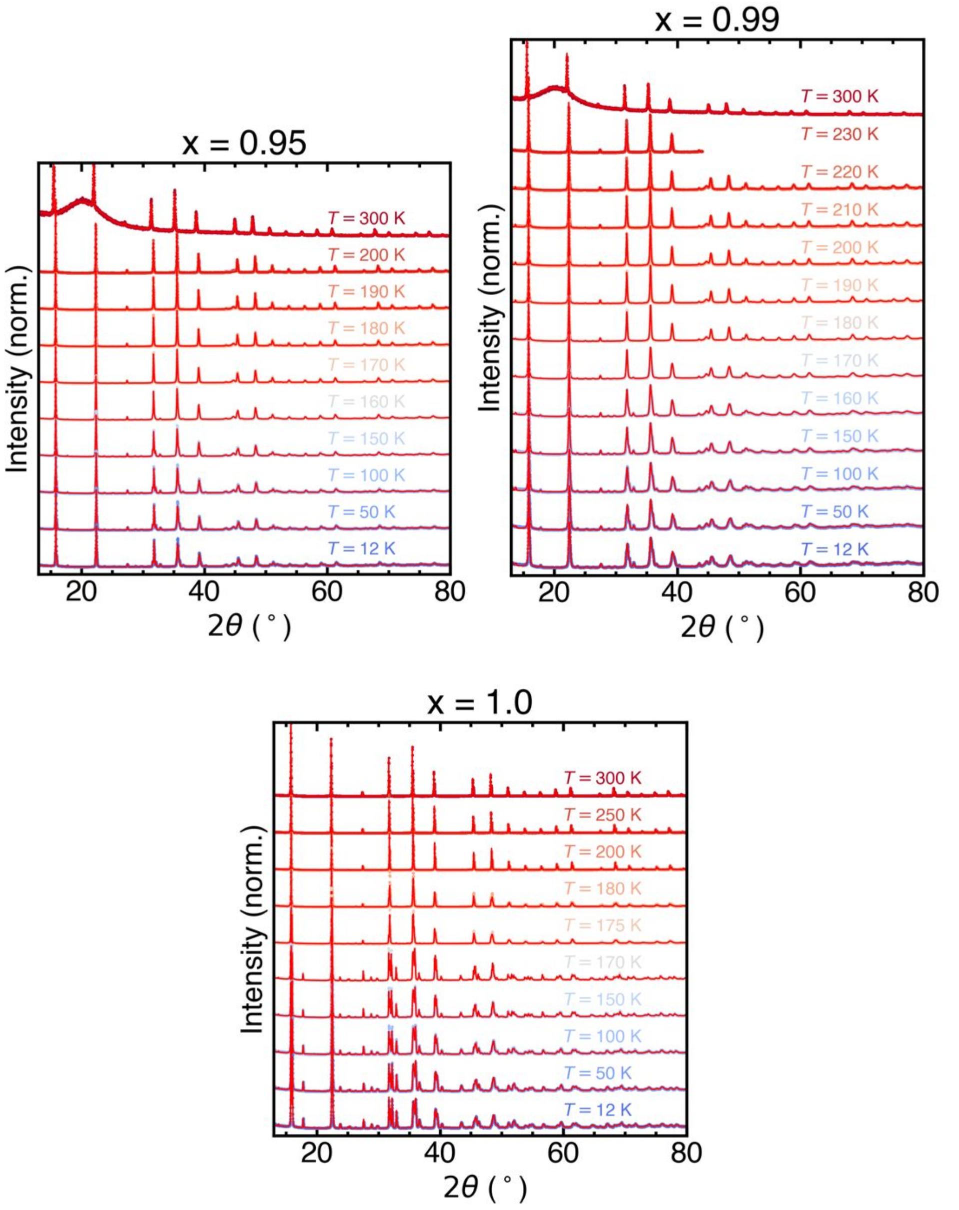

Figure S2 | Le Bail refinements of low temperature X-ray diffraction measurements of $MAPb(Cl_xBr_{1-x})_3$ as a function of the diffraction angle 2θ, temperature *T* and composition *x*. Datapoints are represented by points varying from blue (low temperature) to red (high temperature). Le Bail fits are shown as red lines. The temperatures are given in the same color as the datapoints next to each dataset. All data was collected using the Oxford Cryosystems PheniX, except for the room temperature data of the *x* = 0.2, 0.8, 0.9, 0.95, and 0.99 samples. This was collected using a polymer inert atmosphere holder in the regular configuration of the diffractometer, causing a broad background feature around 2θ = 20°.

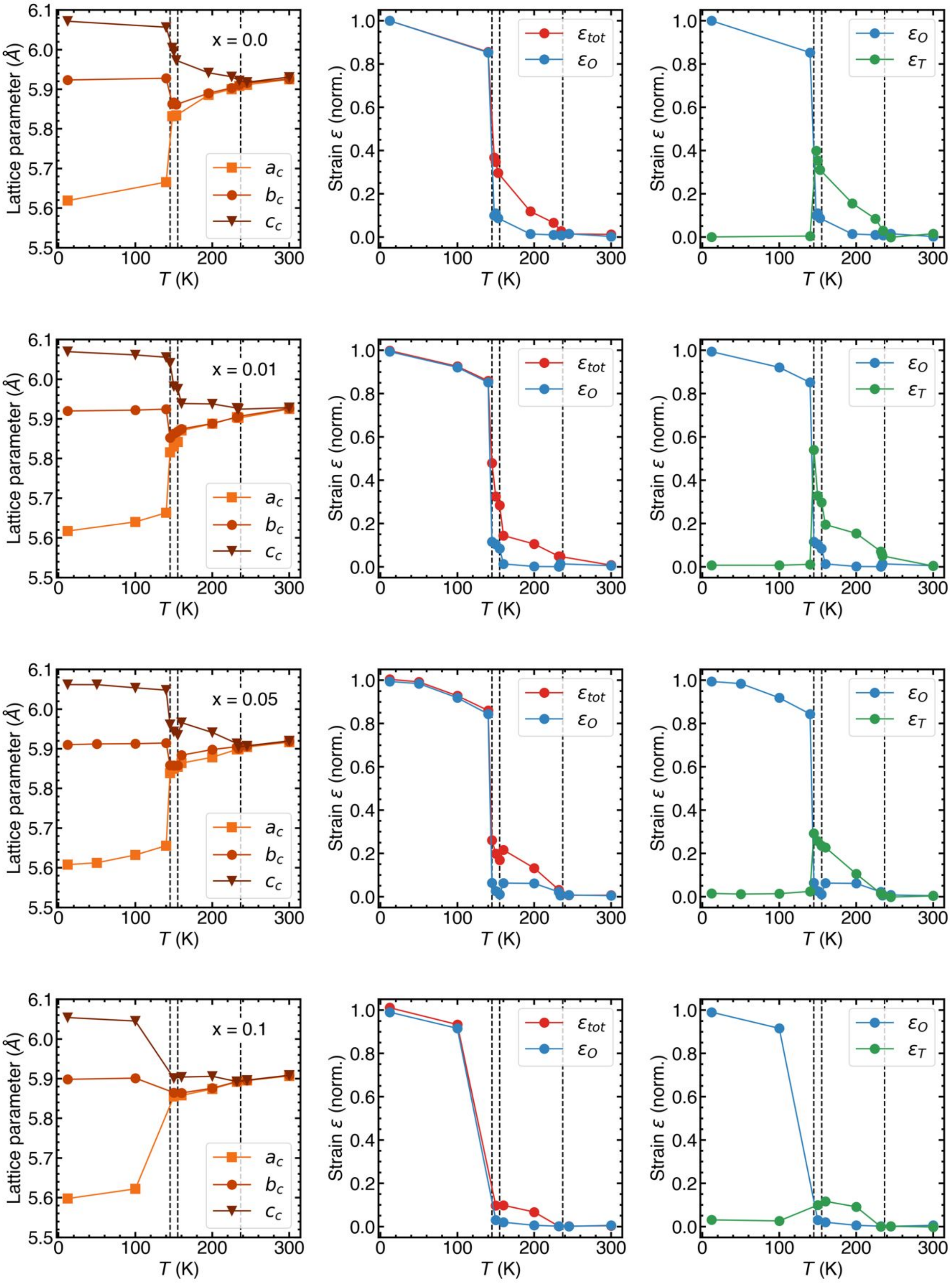
x = 0.0
x = 0.01
x = 0.05
x = 0.1
Lattice parameter (Å)
Strain ε (norm.)
T (K)
a_c
b_c
c_c
ε_{tot}
ε_O
ε_T

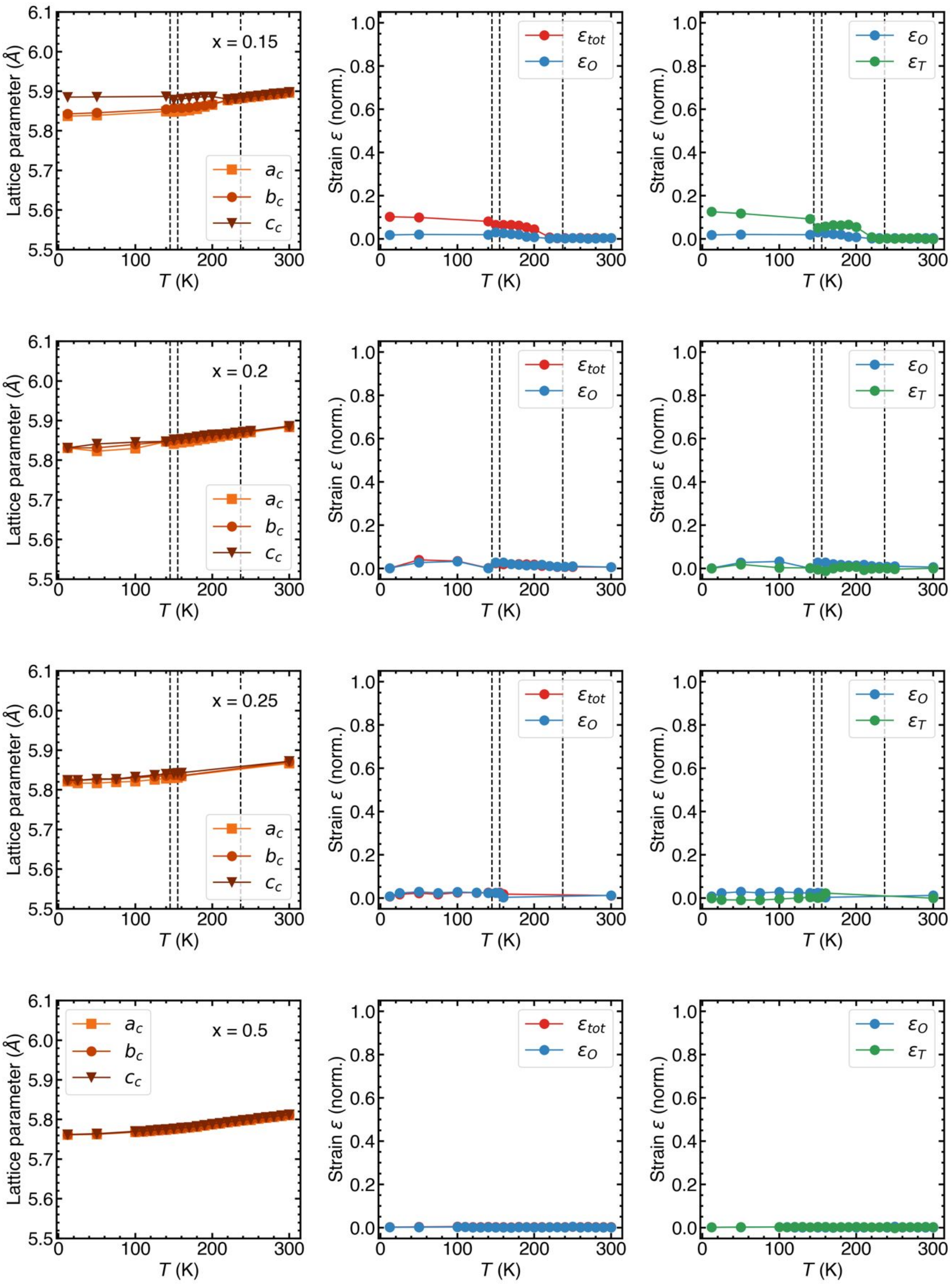
x = 0.15
x = 0.2
x = 0.25
x = 0.5
Lattice parameter (Å)
Strain ε (norm.)
T (K)
a_c
b_c
c_c
ε_{tot}
ε_O
ε_T

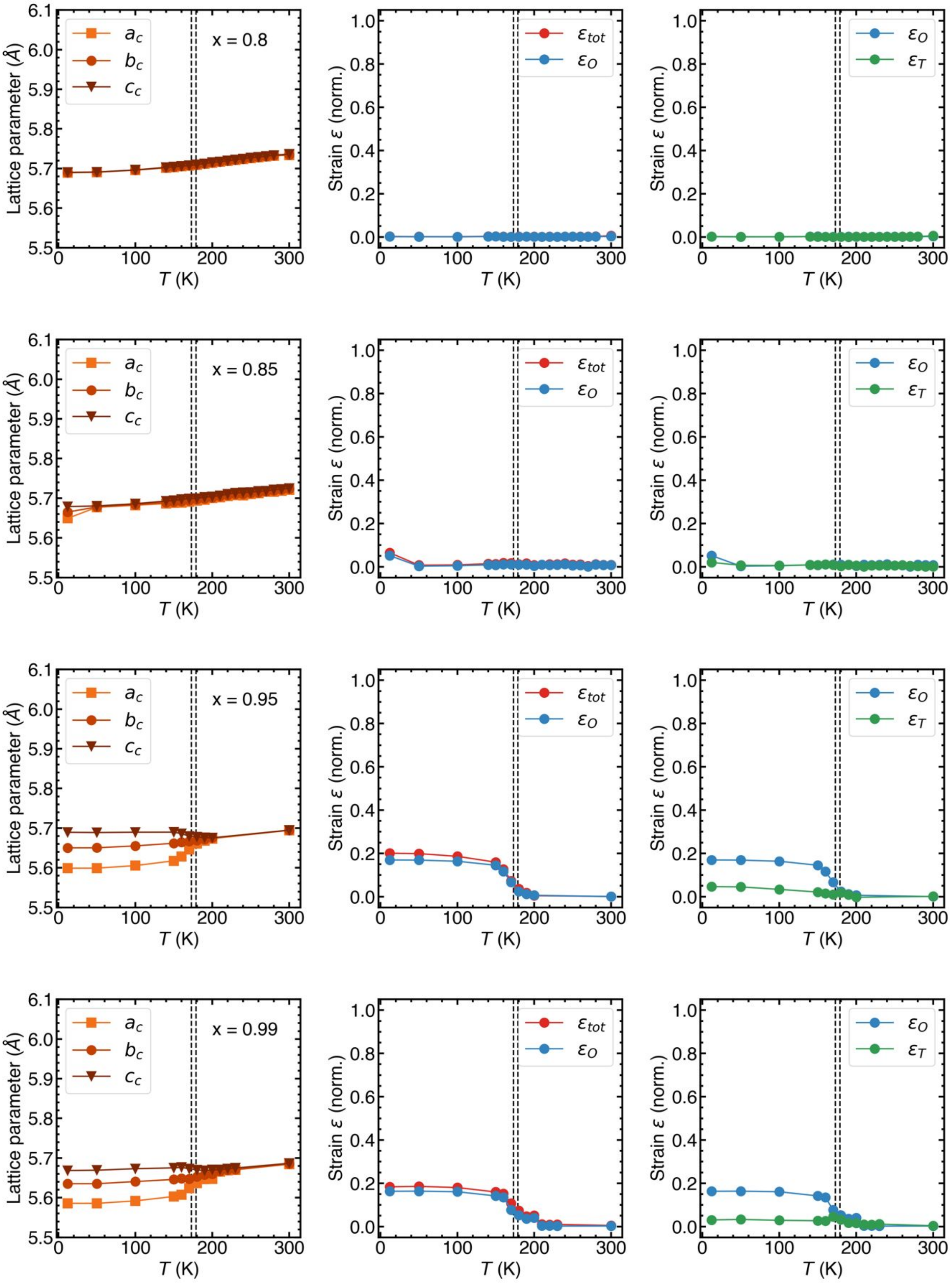

x = 0.8
x = 0.85
x = 0.95
x = 0.99
a_c
b_c
c_c
ε_{tot}
ε_O
ε_T
Lattice parameter (Å)
Strain ε (norm.)
T (K)

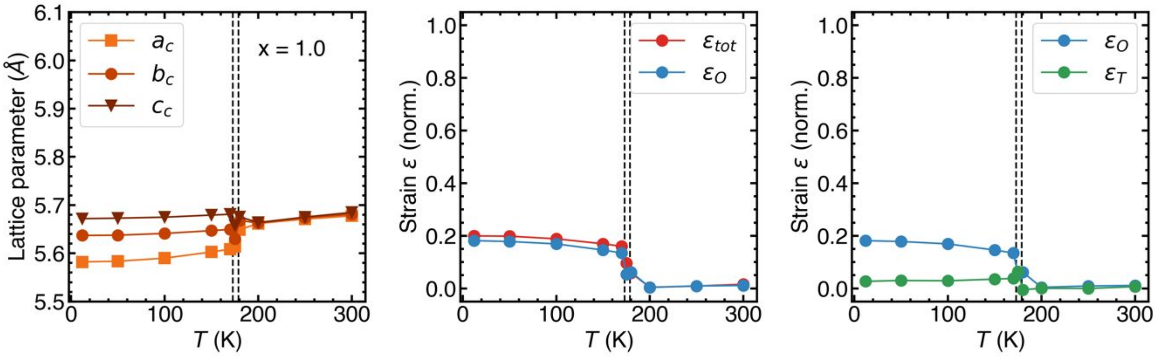

Figure S3 | Pseudocubic lattice parameters of $MAPb(Cl_xBr_{1-x})_3$ extracted from Le Bail[1] fitting of the temperature dependent X-ray diffraction data (left). Distortion parameters calculated from the pseudocubic lattice parameters (middle and right). All distortion parameters are normalised with respect to the largest distortion (x = 0 at T = 12 K). The vertical lines denote the phase transition temperatures of the end member compounds from literature[2].

Section 2. Pseudocubic lattice parameters

We perform Le Bail[1] fitting of the low temperature X-ray diffraction data using the orthorhombic $Pnma$ space group. We use the lattice parameters that have been reported in literature for $MAPbCl_3$[2] and $MAPbBr_3$[3] as a starting point for the fitting of the end members and intermediate compositions. Since both end members feature a different basis for the unit cell, we convert the obtained orthorhombic parameters into pseudocubic lattice parameters to aid the comparison and further analysis. The pseudocubic lattice parameters for $MAPbCl_3$ are defined as follows:

$$a_c = a_o^{Cl}/2,$$

$$b_c = b_o^{Cl}/2,$$

$$c_c = c_o^{Cl}/2,$$

while the pseudocubic lattice parameters for $MAPbBr_3$ are defined as follows:

$$a_c = a_o^{Br}/\sqrt{2},$$

$$b_c = b_O^{Br}/2,$$

$$c_c = c_O^{Br}/\sqrt{2}.$$

Section 3. Low temperature heat capacity fitting

We model the low temperature heat capacity using a linear combination of a single Debye C_D and Einstein C_E term as follows:

$$C_P(T) = DC_D(T) + EC_E(T),$$

where D and E are the magnitude of the contributions of the Debye and Einstein terms, respectively. The Debye term is defined as follows:

$$C_D(T) = 9R\left(\frac{T}{T_D}\right)^3 \int_0^{\frac{T_D}{T}} \frac{x^4 e^x}{e^x - 1} dx,$$

Where R is the universal gas constant and T_D is the Debye temperature. The Einstein term is defined by:

$$C_E(T) = 3R\left(\frac{T_E}{T}\right)^2 \frac{e^{\frac{T_E}{T}}}{\left(e^{\frac{T_E}{T}} - 1\right)^2},$$

where T_E is the Einstein temperature.

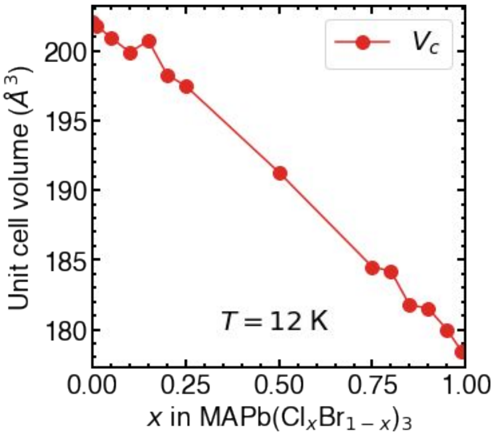

Figure S4 | Pseudocubic unit cell volume of $MAPb(Cl_xBr_{1-x})_3$ as a function of composition at T = 12 K from Le Bail[1] fitting of the X-ray diffraction data with the $Pnma$ structure.

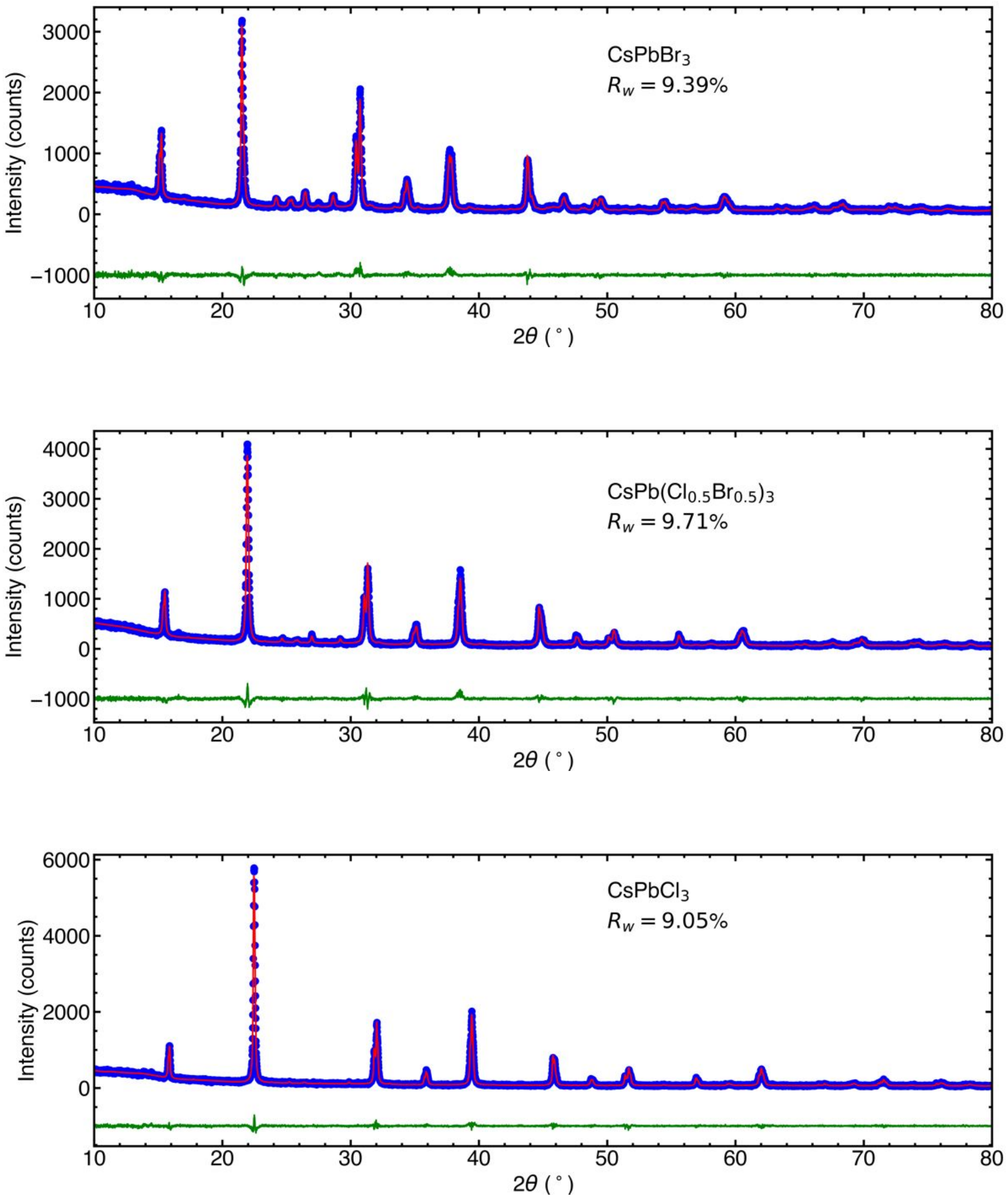

Figure S5 | Room temperature X-ray diffraction data of $CsPb(Cl_xBr_{1-x})_3$ showing intensity as a function of the diffraction angle 2θ, Rietveld[5] refinements are carried out with the Pnma structures of the $CsPbCl_3$ and $CsPbBr_3$ end members from literature[6]. Data is shown as blue circles,

the fits are shown in red and the difference between the data and the fit is shown in green with an offset.

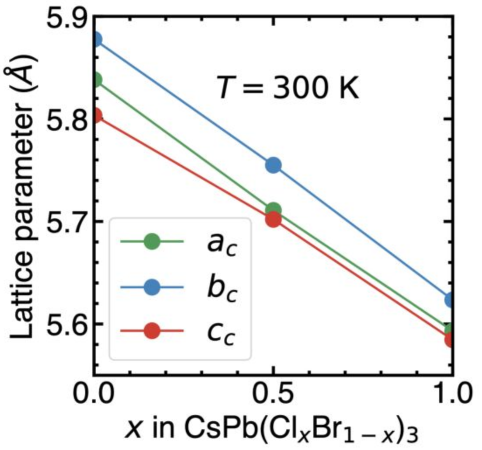

Figure S6 | Pseudocubic lattice parameters of $CsPb(Cl_xBr_{1-x})_3$ obtained from Rietveld[5] refinements of room temperature X-ray diffraction data as a function of composition *x.*

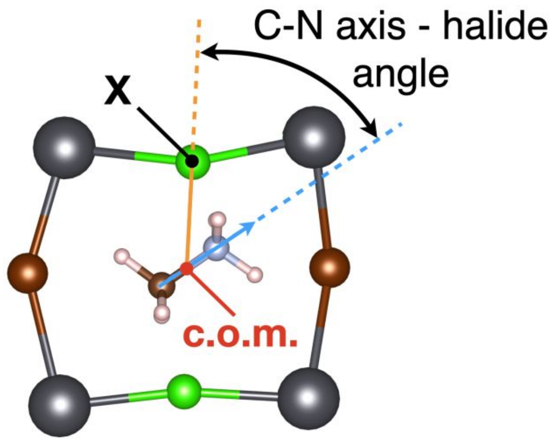

Figure S7 | Schematical drawing illustrating the calculation of the C-N axis - halide angle between the C-N axis of the MA cation (blue line) and the line connecting its center of mass (c.o.m.) with the nearest halide X (orange line).

Section 4. Temperature-dependent photoluminescence linewidth analysis

We here follow the photoluminescence linewidth analysis previously reported for hybrid metal-halide perovskites[7]. The temperature dependent photoluminescence linewidth $\Gamma(T)$ of semiconductors can be attributed to the interaction of charge carriers with phonons. The interaction is described by the following equation:

$$\Gamma(T) = \Gamma_0 + \Gamma_{ac} + \Gamma_{LO} + \Gamma_{imp}$$

$$\Gamma(T) = \Gamma_0 + \gamma_{ac}T + \gamma_{LO}\mathrm{N}_{LO}(T) + \gamma_{imp}e^{-E_b/k_bT},$$

where Γ_0 is the temperature-independent inhomogeneous broadening term related to the scattering from intrinsic disorder in the material[8,9]. The Γ_{ac} and Γ_{LO} terms represent the homogeneous linewidth broadening resulting from acoustic and LO phonon scattering, with charge-carrier coupling strengths γ_{ac} and γ_{LO}, respectively. Electron-phonon coupling is proportional to the occupation number of the phonons, as described by the Bose-Einstein distribution function[10,11]:

$$N_{LO}(T) = \frac{1}{e^{\frac{E_{LO}}{k_BT}} - 1},$$

where E_{LO} is the energy corresponding to the frequency of the LO phonon branch[12,13]. The last term in the equation for photoluminescence linewidth Γ_{imp} accounts for scattering from ionized impurities with an average binding energy E_b that contribute γ_{imp} of broadening when fully ionised. Here we assume $\Gamma_{imp} \approx 0$, supported by observations on the temperature-dependence of the charge-carrier mobility[14–17]. The obtained linewidth and anomalous broadening around *T* = 50 K of the *x* = 0 composition are in agreement with

previous literature[7] (Figure S8a, Table S2). In addition, we here resolve two sudden jumps in linewidth at *T* = 155 K and *T* = 225 K which are close to the phase transitions temperatures for this composition. We do not observe anomalous broadening in the *x* = 0.2 and *x* = 0.5 compositions (Figure S8b, c).

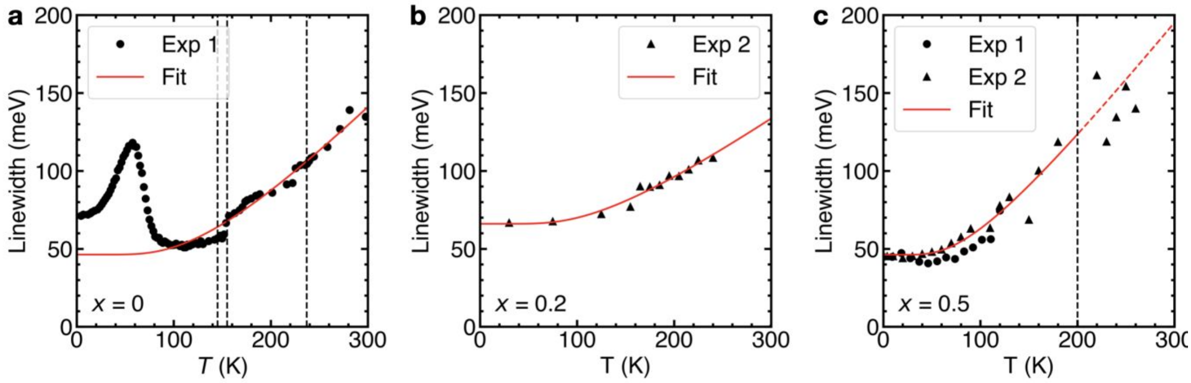

Figure S8 | Photoluminescence linewidth analysis of *x* = 0 (a), *x* = 0.2 (b) and *x* = 0.5 (c) compositions. Data is represented as black dots for experiment 1 and black triangles for experiment 2, fits are shown as red lines. The black vertical dashed lines in (a) represent the phase transition temperatures of *x* = 0 from literature[2], and the onset of photo-induced halide segregation in (c).

Table S2 | Summary of fitted parameters from the PL linewidth analysis.

	Fitting range (K)	Γ_0 (meV)	γ_{LO} (meV)	E_{LO} (meV)
$MAPbBr_3$	80 - 300	46 ± 7	270 ± 104	35 ± 9
$MAPb(Cl_{0.2}Br_{0.8})_3$	10 - 180	66 ± 2	175 ± 74	33 ± 7
$MAPb(Cl_{0.5}Br_{0.5})_3$	4 - 200	46 ± 3	201 ± 124	22 ± 7

Refences

(1) Le Bail, A.; Duroy, H.; Fourquet, J. L. Ab-Initio Structure Determination of LiSbWO6 by X-Ray Powder Diffraction. *Mater. Res. Bull.* **1988**, *23* (3), 447–452. https://doi.org/https://doi.org/10.1016/0025-5408(88)90019-0.

(2) Onoda-Yamamuro, N.; Matsuo, T.; Suga, H. Calorimetric and IR Spectroscopic Studies of Phase Transitions in Methylammonium Trihalogenoplumbates (II). *J. Phys. Chem. Solids* **1990**, *51* (12), 1383–1395. https://doi.org/10.1016/0022-3697(90)90021-7.

(3) Chen, K.; Deng, X.; Goddard, R.; Tüysüz, H. Pseudomorphic Transformation of Organometal Halide Perovskite Using the Gaseous Hydrogen Halide Reaction. *Chem. Mater.* **2016**, *28* (15), 5530–5537. https://doi.org/10.1021/acs.chemmater.6b02233.

(4) Swainson, I. P.; Hammond, R. P.; Soullière, C.; Knop, O.; Massa, W. Phase Transitions in the Perovskite Methylammonium Lead Bromide, CH3ND3PbBr3. *J. Solid State Chem.* **2003**, *176* (1), 97–104. https://doi.org/10.1016/S0022-4596(03)00352-9.

(5) Rietveld, H. M. A Profile Refinement Method for Nuclear and Magnetic Structures. *J. Appl. Crystallogr.* **1969**, *2* (2), 65–71. https://doi.org/10.1107/s0021889869006558.

(6) Linaburg, M. R.; McClure, E. T.; Majher, J. D.; Woodward, P. M. Cs1-XRbxPbCl3 and Cs1-XRbxPbBr3 Solid Solutions: Understanding Octahedral Tilting in Lead Halide Perovskites. *Chem. Mater.* **2017**, *29* (8), 3507–3514. https://doi.org/10.1021/acs.chemmater.6b05372.

(7) Wright, A. D.; Verdi, C.; Milot, R. L.; Eperon, G. E.; Pérez-Osorio, M. A.; Snaith, H. J.; Giustino, F.; Johnston, M. B.; Herz, L. M. Electron-Phonon Coupling in Hybrid Lead Halide Perovskites. *Nat. Commun.* **2016**, *7* (May). https://doi.org/10.1038/ncomms11755.

(8) Rudin, S.; Reinecke, T. L.; Segall, B. Temperature-Dependent Exciton Linewidths in

Semiconductors. *Phys. Rev. B* **1990**, *42* (17), 218–231.

(9) Malikova, L.; Krystek, W.; Pollak, F. H.; Dai, N.; Cavus, A.; Tamargo, M. C. Temperature Dependence of the Direct Gaps of ZnSe and Zn0.56Cd0.44Se. *Phys. Rev. B* **1996**, *54* (3), 1819–1824. https://doi.org/10.1103/PhysRevB.54.1819.

(10) Bartolo, B. D. & Chen, X. *Advances in Energy Transfer Processes*; 2001.

(11) Chen, Y.; Kothiyal, G. P.; Singh, J.; Bhattacharya, P. K. Absorption and Photoluminescence Studies of the Temperature Dependence of Exciton Life Time in Lattice-Matched and Strained Quantum Well Systems. *Superlattices Microstruct.* **1987**, *3* (6), 657–664. https://doi.org/https://doi.org/10.1016/0749-6036(87)90195-9.

(12) Selci, S.; Cricenti, A.; Righini, M.; Petrillo, C.; Sacchetti, F.; Alexandre, F.; Chiarotti, G. Evaluation of Electron-Phonon Coupling of Al0.27Ga0.73As/GaAs Quantum Wells by Normal Incidence Reflectance. *Solid State Commun.* **1991**, *79* (7), 561–565. https://doi.org/https://doi.org/10.1016/0038-1098(91)90910-N.

(13) YU, P.; Cardona, M. *Fundamentals of Semiconductors: Physics and Materials Properties*; Graduate Texts in Physics; Springer Berlin Heidelberg, 2010.

(14) Milot, R. L.; Eperon, G. E.; Snaith, H. J.; Johnston, M. B.; Herz, L. M. Temperature-Dependent Charge-Carrier Dynamics in CH3NH3PbI3 Perovskite Thin Films. *Adv. Funct. Mater.* **2015**, *25* (39), 6218–6227. https://doi.org/https://doi.org/10.1002/adfm.201502340.

(15) Oga, H.; Saeki, A.; Ogomi, Y.; Hayase, S.; Seki, S. Improved Understanding of the Electronic and Energetic Landscapes of Perovskite Solar Cells: High Local Charge Carrier Mobility, Reduced Recombination, and Extremely Shallow Traps. *J. Am. Chem. Soc.* **2014**, *136* (39), 13818–13825. https://doi.org/10.1021/ja506936f.

(16) Savenije, T. J.; Ponseca, C. S.; Kunneman, L.; Abdellah, M.; Zheng, K.; Tian, Y.; Zhu, Q.; Canton, S. E.; Scheblykin, I. G.; Pullerits, T.; Yartsev, A.; Sundström, V. Thermally Activated Exciton Dissociation and Recombination Control the Carrier Dynamics in Organometal Halide Perovskite. *J. Phys. Chem. Lett.* **2014**, *5* (13), 2189–2194. https://doi.org/10.1021/jz500858a.

(17) Karakus, M.; Jensen, S. A.; D'Angelo, F.; Turchinovich, D.; Bonn, M.; Cánovas, E. Phonon–Electron Scattering Limits Free Charge Mobility in Methylammonium Lead Iodide Perovskites. *J. Phys. Chem. Lett.* **2015**, *6* (24), 4991–4996. https://doi.org/10.1021/acs.jpclett.5b02485.
